# Enzymatic independent role of sphingosine kinase 2 in regulating the expression of type I interferon during influenza A virus infection

**DOI:** 10.1371/journal.ppat.1010794

**Published:** 2022-09-07

**Authors:** Mengqiong Xu, Sisi Xia, Mei Wang, Xiaolian Liu, Xin Li, Weijie Chen, Yaohao Wang, Hongjian Li, Chuan Xia, Jun Chen, Jianguo Wu

**Affiliations:** 1 Guangdong Provincial Key Laboratory of Virology, Institute of Medical Microbiology, Jinan University, Guangzhou, China; 2 Department of Biotechnology, College of Life Science and Technology, Jinan University, Guangzhou, China; 3 Department of Biological Engineering, Wuhan Polytechnic University, Wuhan, China; 4 College of Basic Medical Sciences, Dalian Medical University, Dalian, China; 5 Foshan Institute of Medical Microbiology, Foshan, Guangdong, China; University of Maryland, UNITED STATES

## Abstract

Influenza virus has the ability to circumvent host innate immune system through regulating certain host factors for its effective propagation. However, the detailed mechanism is still not fully understood. Here, we report that a host sphingolipid metabolism-related factor, sphingosine kinase 2 (SPHK2), upregulated during influenza A virus (IAV) infection, promotes IAV infection in an enzymatic independent manner. The enhancement of the virus replication is not abolished in the catalytic-incompetent SPHK2 (G212E) overexpressing cells. Intriguingly, the sphingosine-1-phosphate (S1P) related factor HDAC1 also plays a crucial role in SPHK2-mediated IAV infection. We found that SPHK2 cannot facilitate IAV infection in HDAC1 deficient cells. More importantly, SPHK2 overexpression diminishes the IFN-β promoter activity upon IAV infection, resulting in the suppression of type I IFN signaling. Furthermore, ChIP-qPCR assay revealed that SPHK2 interacts with IFN-β promoter through the binding of demethylase TET3, but not with the other promoters regulated by TET3, such as TGF-β1 and IL6 promoters. The specific regulation of SPHK2 on IFN-β promoter through TET3 can in turn recruit HDAC1 to the IFN-β promoter, enhancing the deacetylation of IFN-β promoter, therefore leading to the inhibition of IFN-β transcription. These findings reveal an enzymatic independent mechanism on host SPHK2, which associates with TET3 and HDAC1 to negatively regulate type I IFN expression and thus facilitates IAV propagation.

## Author summary

Sphingosine-1-phosphate (S1P) metabolizing related proteins are important regulators in response to the infection and pathogenicity of influenza virus and may serve as potential anti-viral targets. Our previous study identifies SPHK2 as a positive regulator of IAV replication, the present study demonstrates that IAV can increase the expression of SPHK2 which in turn diminishes the type I IFN mediated antiviral responses through HDAC1 and TET3. The study highlights a subtle non-enzymatic mechanism of SPHK2 in inhibiting the transcription of type I interferon, and reveals how IAV evade host immunity and replicate in host cells with the help of SPHK2, giving us a novel insight into the interplay between IAV and host immunity.

## Introduction

Pandemic influenza viruses, leading to severe acute respiratory syndromes, result in immense health risks and great economic losses worldwide owing to the highest rates of morbidity and fatality to humans and animals during each influenza season [1,2]. IAV infection in certain groups are more severe than it in others because of individual variation in immune responses (susceptibility, duration, and intensity), which may be associated with host genetic factors that influence viral replication [3]. Multiple genome-wide screening approaches applied by different studies have also identified a myriad of host factors that may have pro-viral effect on IAV infection [4–7]. Our previous research has showed that poly (ADP-ribose) polymerase 1 (PARP1), a cellular protein that its interaction with hemagglutinin (HA) of influenza A virus is originally identified by mass spectrometry analysis, has been proved to facilitate viral propagation via mediating the IAV HA-induced reduction of IFNAR1 [8,9]. These host factors have been described to be necessary for virus entry, genomic replication, or negative regulation of cell-intrinsic immunity at various steps of IAV infection. However, the exact mechanisms are still lacking for most of the IAV-related host cell factors. As genetic mutation and drift of viral genome in several different strains occurs at an ever-increasing rate during each flu season, the better understanding of these factors not only informs us of the individual immune differences in response to IAV infection, but also provides alternative therapeutic strategies and ideas for anti-viral drug design.

Sphingosine-1-phosphate (S1P), an important regulator of inflammation and immune responses, engages in the immune cells-trafficking through sharp gradient between the levels of S1P in the circulation and those in the tissues. S1P synthesized by sphingosine after phosphorylation is catalyzed by sphingosine kinase, SPHK1 and SPHK2 [10]. Both of them exert various biological and physiological functions through binding to the five tightly regulated spatial and temporal expressed sphingosine-1-phosphate receptor subfamily comprising five members (S1PR1-5), with activation of various signaling cascades [10,11]. Besides, S1P produced by cytoplasmic membrane SPHK1, a cofactor for TNF receptor-associated factor 2 (TRAF2), has important intracellular function, motivating TRAF2-mediated ubiquitylation of receptor-interacting serine/threonine protein kinase 1 (RIP1) and leading to the activation of NF-κB [12]. Therefore, it leaves us wondering whether metabolizing related proteins of S1P have significant role on restricting the infection and pathogenicity of influenza virus and may serve as potential anti-viral targets. We have found S1P lyase (SPL), which results in the irreversible cleavage of S1P, can protect cells from IAV infection through the activation of JAK/STAT pathway, while sphingosine kinases that produce S1P make the cultured cells and mice more susceptible to IAV infection [13,14]. Furthermore, we have proved that sphingosine kinase 1 (SPHK1) is served as a favorable factor for viral replication by activating the NF-κB signaling to support viral RNA synthesis and regulating the nuclear export of vRNP during IAV infection [15]. Yet, as another isoenzyme of sphingosine kinase, the specific immune regulation mechanism of SPHK2 on promotion of IAV propagation still needs to be elucidated.

Although SPHK1 and SPHK2 share high homology of amino acid sequence with each other, they have overlapping but distinct functions. Studies have reported that SPHK1 mainly localizes to the cytosol and plasma membrane; in contrast, SPHK2 is found to shuttle between the nucleus, nucleoplasm and cytoplasm, but it cannot be ruled out to function in the endoplasmic reticulum [16]. In addition, SPHK1 is verified to activate TNF-α and NF-κB signaling in response to inflammation, promoting cell survival and proliferation, whereas SPHK2 has been controversially reported to stimulate cell apoptosis [16,17]. Especially, Nitai C. Hait *et al*. have revealed that SPHK2 is assembled in corepressor complexes containing histone deacetylase 1 (HDAC1) and the overexpression of SPHK2 leads to nuclear S1P accumulation, which inhibits HDAC1 activity, thereby causes increased acetylation of histone lysine residues and subsequently activates p21 and c-fos gene transcription [18]. Recently, it has also been reported that inhibition of SPHK2 can enhance protective T cell immune responses and gradually terminate lymphocytic choriomeningitis virus Cl 13 (LCMV Cl 13) persistence in mice [19]. SPHK2 colocalizes with chikungunya virus (CHIKV) RNA and nonstructural proteins, and impairment of SPHK2 expression can significantly inhibit CHIKV infection [20]. Inhibition of SPHK2 also limits HIV-1 infection in CD4 T cells [21]. Moreover, inhibition of SPHK2 induced viral lytic gene expression with KSHV-infected endothelial cells, indicating the function of SPHK2 in maintaining viral latency [22]. Interestingly, it has been reported that SPHK2 functions differently during the infection of different genotypes of HCV. The cell-cultured adapted genotype 1 HCVs in Huh-7.5 cells can be propagated by the SPHKs inhibitor, SKI, when its concentration set to selectively inhibit SPHK2, however, a chimeric genotype 2 virus (HJ3-5/GLuc) of HCVs is not suppressed after SKI treatment [23]. In view of the complex functions of SPHK2 in response to virus infection, the role of the SPHK2 protein itself and the S1P-producing kinase activity of SPHK2 in regulation of IAV replication should be evaluated.

HDAC1, as the most widely studied deacetylase, plays a significant role in epigenetic regulation of cellular genes and serves as an important regulator of innate immunity during viral infection [24–26]. Porcine epidemic diarrhea virus (PEDV) inhibits the expression of HDAC1 through the interaction of its N protein with the transcription factor Sp1, therefore facilitating the viral replication [27]. However, inhibition of HDAC1 suppresses pseudorabies virus (PRV) infection through cGAS-STING antiviral innate immunity [28]. Transient knockdown of HDAC1 also suppresses HIV-1 replication [29]. Moreover, HIV-1 integrase (IN) and cellular protein INI1/hSNF5 bind SIN3 Associated Protein 18 (SAP18) and recruit components of Sin3a-HDAC1 complex into HIV-1 virions to facilitate the viral replication [30]. Conversely, HDAC1, in complex with KAP1 (TRIM28) can deacetylate HIV-1 IN, downregulating the efficiency of HIV-1 integration [31]. These studies indicate that the functions of HDAC1 on the regulation of viral innate immunity are much more complicated.

Ten-eleven translocation protein 3 (TET3), a member of dioxygenases family, induces cytosine demethylation by converting 5-methylcytosine (5mC) to 5-hydroxymethylcytosine (5hmC) at target gene promoters, thereby creating a global and local dynamic methylation landscape in epigenetic expression under specific physiological conditions [32,33]. For example, TET3 in B cells is needed to repress CD86 and prevent autoimmunity [34]. TET3 increases IL6 expression by up-regulation of 5hmC in IL6 promoter in chronic hypoxia induced atherosclerosis in offspring rats [35]. TET3 and TGF-β1 form a positive feedback loop to promote TGF-β signaling and subsequently profibrotic gene expression in hepatic stellate cells (HSCs) [36]. TET3 also demethylates the gene promoter of miR-30d precursor to block TGF-β1-induced epithelial-mesenchymal transition (EMT), thus can act as a suppressor of ovarian cancer [37].

In this study, we explored the potential molecular mechanisms of SPHK2 in influenza A virus infection. We substantiated that, IAV manipulates SPHK2 to subvert host type I IFN responses to gain a better replication. However, SPHK2 catalytic-inactive mutant does not lose the ability to enhance the IAV replication, suggesting the pro-viral function of SPHK2 is independent of its enzymatic activity. Furthermore, we proved that the inhibitory effect on IAV-induced type I IFN transcription by SPHK2 is dependent on HDAC1 and the specific IFN-β promoter binding demethylase TET3 [38]. The study reveals a subtle non enzymatic mechanism of SPHK2 in downregulating the expression of type I interferon upon IAV infection.

## Results

### Influenza virus infection increases the expression of *SPHK2*

**Fig 1:**
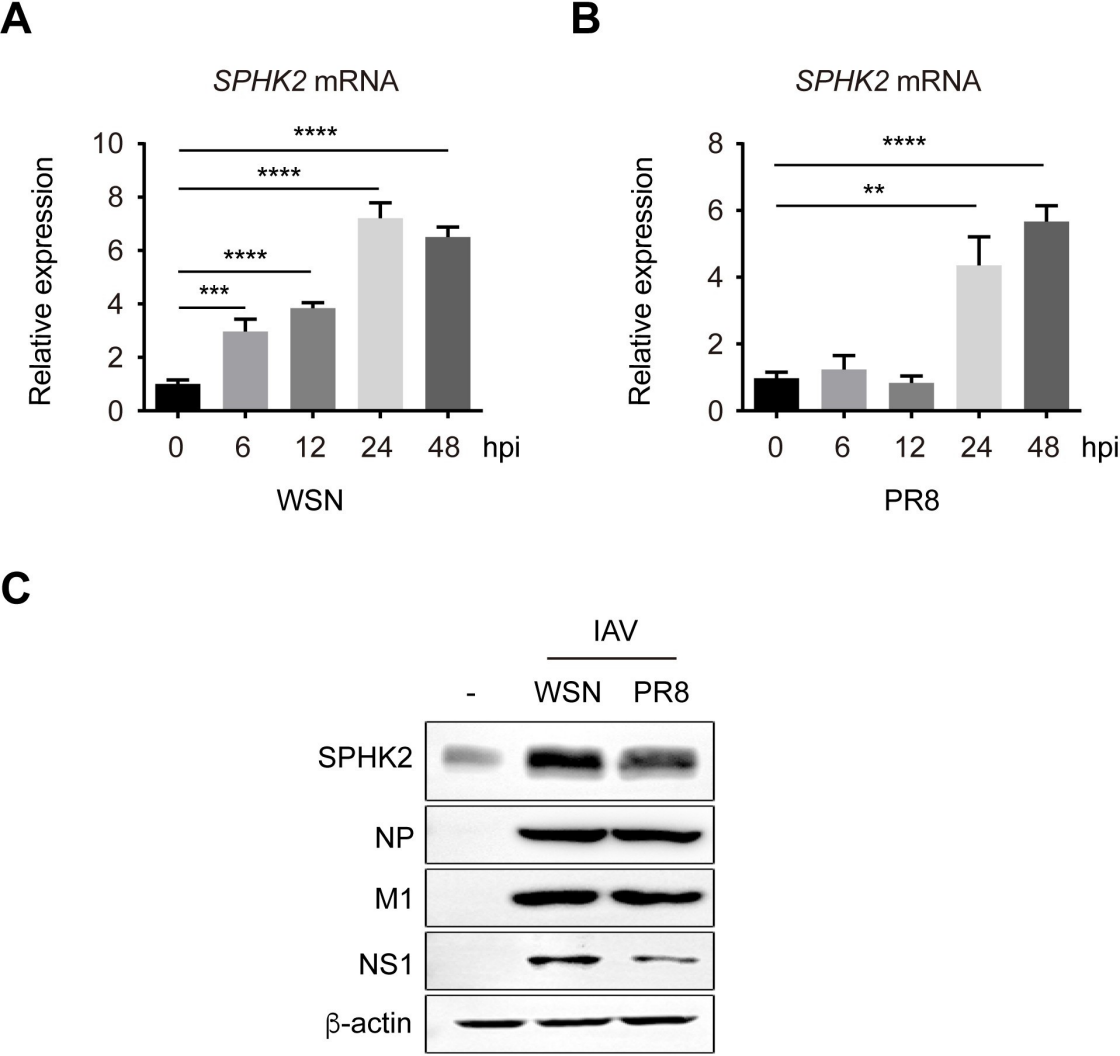
IAV increases the mRNA levels of SPHK2. (**A**) A549 cells were infected with influenza A/WSN/33 (H1N1) virus (WSN) or (**B**) influenza A/PR/8/34 (H1N1) virus (PR8) at an MOI of 0.5 for the indicated times. The mRNA levels of SPHK2 were analyzed by qRT-PCR at 0, 6, 12, 24 and 48 hpi, which was normalized to GAPDH. Error bars represent mean values ± SD calculated from the results for three individual samples. **, P<0.01; ***, P<0.001; ****, P<0.0001. (**C**) A549 cells were infected with influenza A/WSN/33 (H1N1) virus (WSN) or influenza A/PR/8/34 (H1N1) virus (PR8) at an MOI of 0.5 for 24 h. The protein level of SPHK2 was measured by Western blotting. NP, M1 and NS1 were used as positive controls of IAV infection, and β-actin was an internal loading control.

Viruses can subvert host cell transcriptional or translational machinery to express their own polypeptides and/or regulate the expression of host protein in favor of virus replication [39]. We have previously reported that influenza virus infection upregulates the protein levels of SPHK2 [14]. However, whether the mRNA levels of SPHK2 are also affected by virus is not clear. Consequently, we first measured the mRNA expression of SPHK2 in infected A549 cells. We found that both WSN and PR8 viruses upregulated the SPHK2 mRNA levels. IAV WSN infection led to statistically significant (6 h, P<0.001; 12 h, P<0.0001; 24 h, P<0.0001; 48 h, P<0.0001) increase in SPHK2 mRNA levels in A549 cells (Fig 1A). The same results (24 h, P<0.01; 48 h, P<0.0001) were also obtained in IAV PR8 infected cells (Fig 1B). However, the change of SPHK2 mRNA levels in PR8 infected A549 cells was slower than that in WSN infected A549 cells, which might be caused by different replication kinetics or cytopathic effects among various strains, and the specific reasons underlying this phenomenon need to be further investigated. Next, the levels of SPHK2 protein were detected in A549 cells infected with IAV. We observed that compared to uninfected A549 cells, SPHK2 protein level in IAV infected A549 cells was elevated which is in accordance with our previous findings [14]. We noticed that the SPHK2 protein level in WSN infected A549 cells as well as in PR8 infected A549 cells was obviously increased (Fig 1C). These results suggest that IAV infection induces SPHK2 upregulation at the mRNA level.

### Influenza virus infection induces the nuclear accumulation and translocation of SPHK2

**Fig 2:**
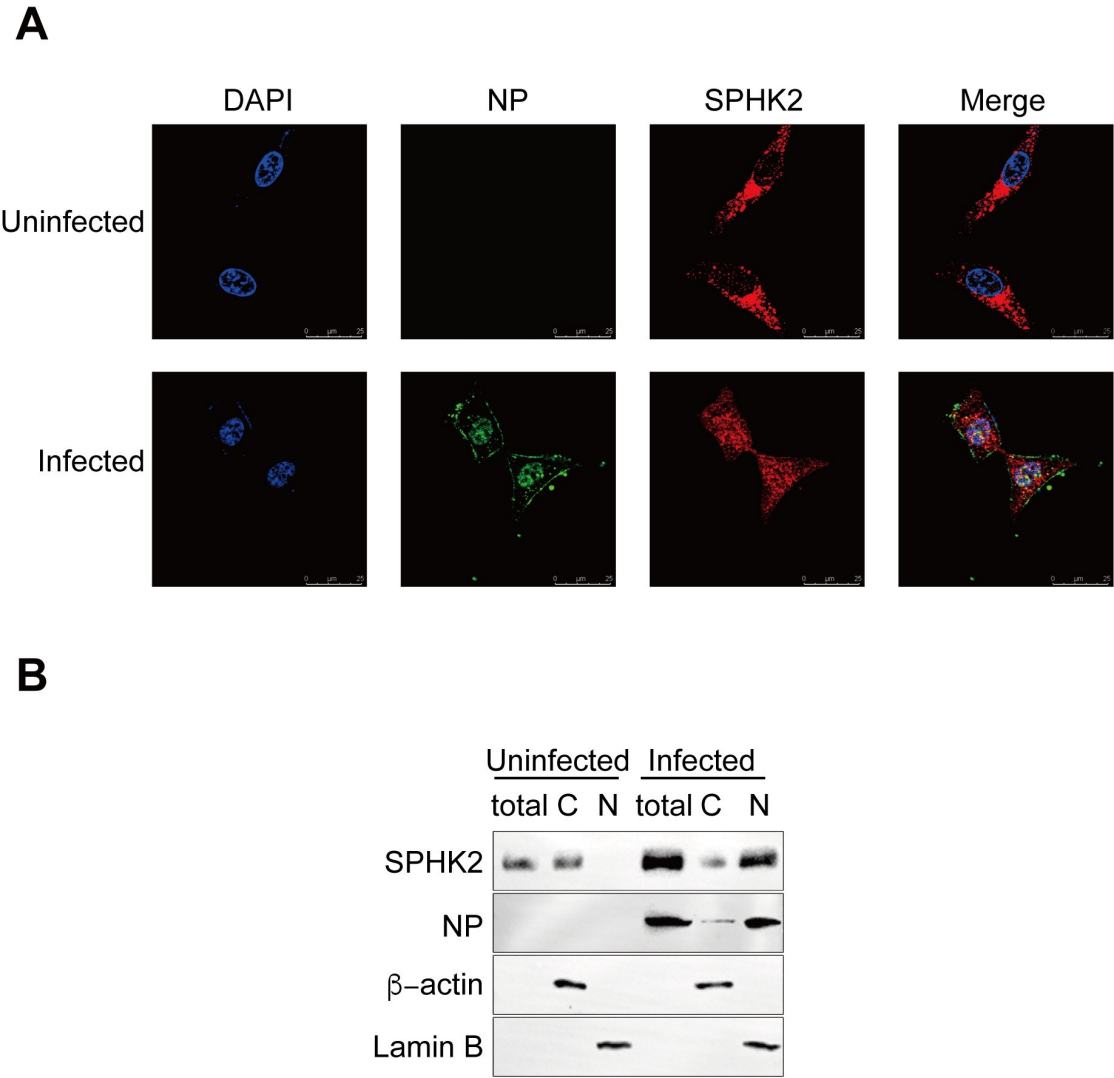
IAV affects SPHK2’ nucleocytoplasmic distribution. A549 cells were uninfected or infected with influenza A/WSN/33 (H1N1) virus (WSN) at an MOI of 0.5 for 6 h. (**A**) Cells were fixed and stained using DAPI for nuclei (Blue), as well as anti-influenza NP (Green) and anti-SPHK2 antibodies (Red). The cells were visualized by confocal laser scanning microscopy. (**B**) The protein levels of SPHK2 and IAV NP in uninfected or infected A549 cells were detected by Western blotting, β-actin and Lamin B were used as loading controls for cytoplasmic and nuclear proteins, respectively. Results are representatives of three independent experiments.

Since IAV infection leads to the accumulation of SPHK2, we then measured the effects of IAV on the localization of SPHK2 in A549 cell lines by immunostaining studies. Endogenous SPHK2 proteins were found mainly located in the cytoplasm, with only a small amount of these proteins present in the nucleus of mock-infected A549 cells. However, after 6 h of infection with IAV, SPHK2 were distributed not only in the cytoplasm, but also in the nucleus, and displayed an obvious accumulation in the nucleus (Fig 2A). Western blotting assay also indicated that IAV induced the nuclear accumulation of SPHK2 in A549 cells (Fig 2B). We found that there was still a large amount of SPHK2 accumulation maintaining in the nucleus at 24 h post-infection with IAV. However, we didn’t find any distinguishable increased expression or accumulation of SPHK2 in the nucleus when tested with ZIKV or EV71 infection (S1A and S1B Fig). These findings demonstrate that IAV infection increases the nuclear accumulation and translocation of SPHK2, and the accumulation of SPHK2 in the nucleus was specific to influenza A virus infection.

### SPHK2 has a pro-viral effect on influenza virus replication

**Fig 3:**
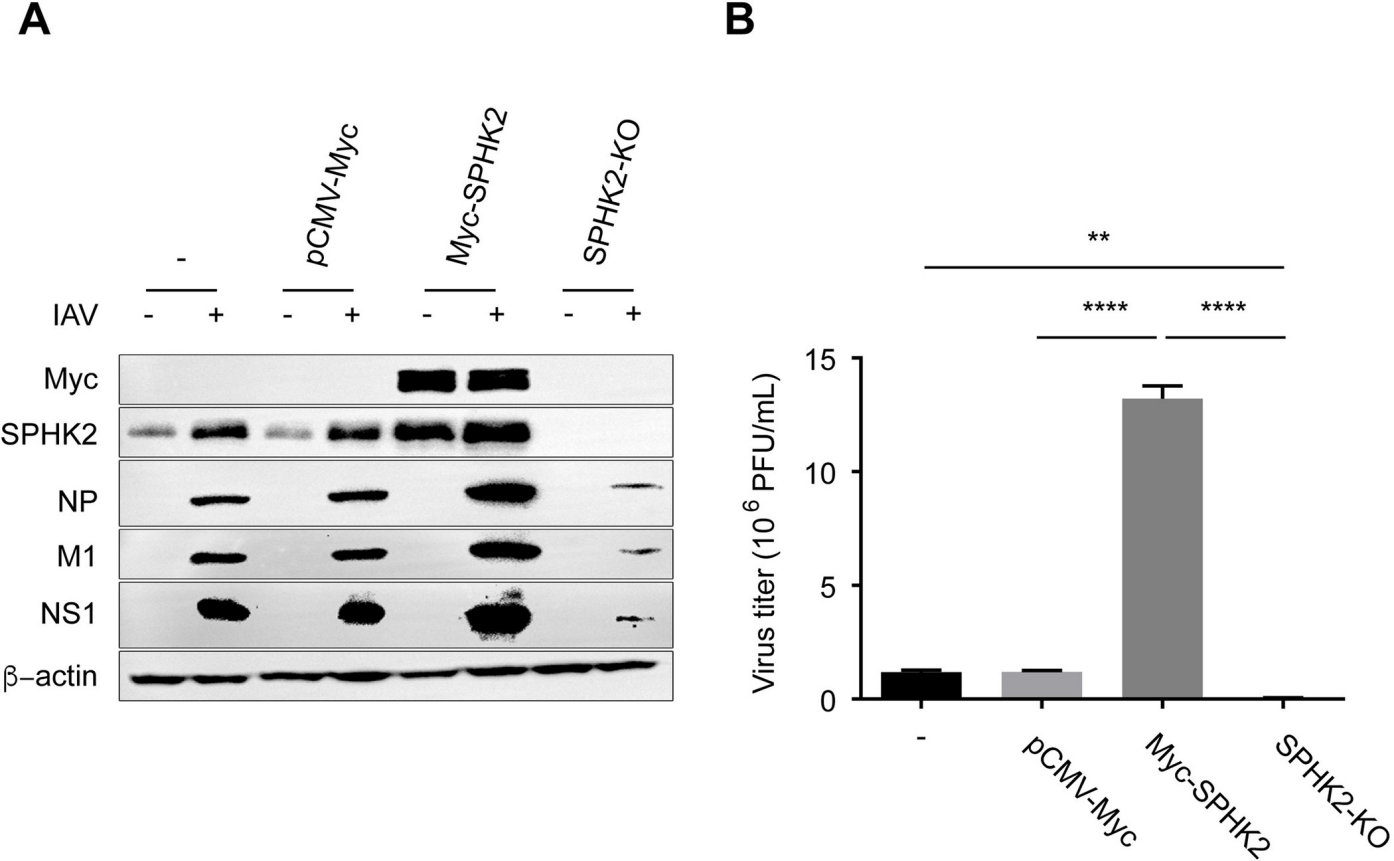
SPHK2 facilitates IAV replication. (**A**) A549 cells were transfected with plasmids encoding SPHK2 or a control vector pCMV-Myc, and 24 h after transfection, cells were infected with WSN virus at an MOI of 0.5. A549 cells or sgSPHK2-1 A549 cells were infected with WSN virus at an MOI of 0.5. At 24 hpi, Western blotting analysis was performed to detect viral NP, M1, NS1 and β-actin is shown as a loading control. (**B**) Virus titers in IAV (MOI = 0.5) infected A549 cells transfected with the plasmids pCMV-myc or Myc-SPHK2 and virus titers in IAV infected A549 cells or IAV infected sgSPHK2-1 A549 cells was determined in MDCK cells by plaque assay. Values are means ± SD (n = 3). Data are representative of three independent experiments. **, P<0.01; ****, P<0.0001.

Based on the above findings, we hypothesized that IAV could regulate host SPHK2 to obtain a better replication in host cells. To confirm the function of SPHK2, A549 cells were overexpressed with Myc-tagged SPHK2, and the effect of SPHK2 overexpression on A549 cell growth rate was detected (S2A Fig). To further confirm the importance of SPHK2 in influenza virus replication, we utilized CRISPR-Cas9 system to generate clonal SPHK2 knockout A549 cells. Western blotting and qPCR analysis for endogenous SPHK2 revealed a dramatic reduction in SPHK2 protein levels in sgSPHK2-1 cells (S2B Fig). Knockout of SPHK2 also has no influence on the growth rate of constructed cells (S2C Fig). Thus, the sgSPHK2-1 A549 cell line was selected for the subsequent experiments. SPHK2 overexpression was found to increase the protein level of viral M1 and NS1, however cells lacking SPHK2 exhibited significant reduction in viral protein synthesis (Fig 3A). Moreover, virus titers in SPHK2 overexpressed A549 cells were increased by approximately 1.2-log (P<0.0001) at 24 h post infection, while the viral titers in SPHK2 knockout A549 cells dropped more than 1.3-log (P<0.01), compared to that in the WT A549 cells (Fig 3B). Thus, these results indicate that SPHK2 facilitates IAV infection.

### SPHK2 regulates influenza virus infection through HDAC1

**Fig 4:**
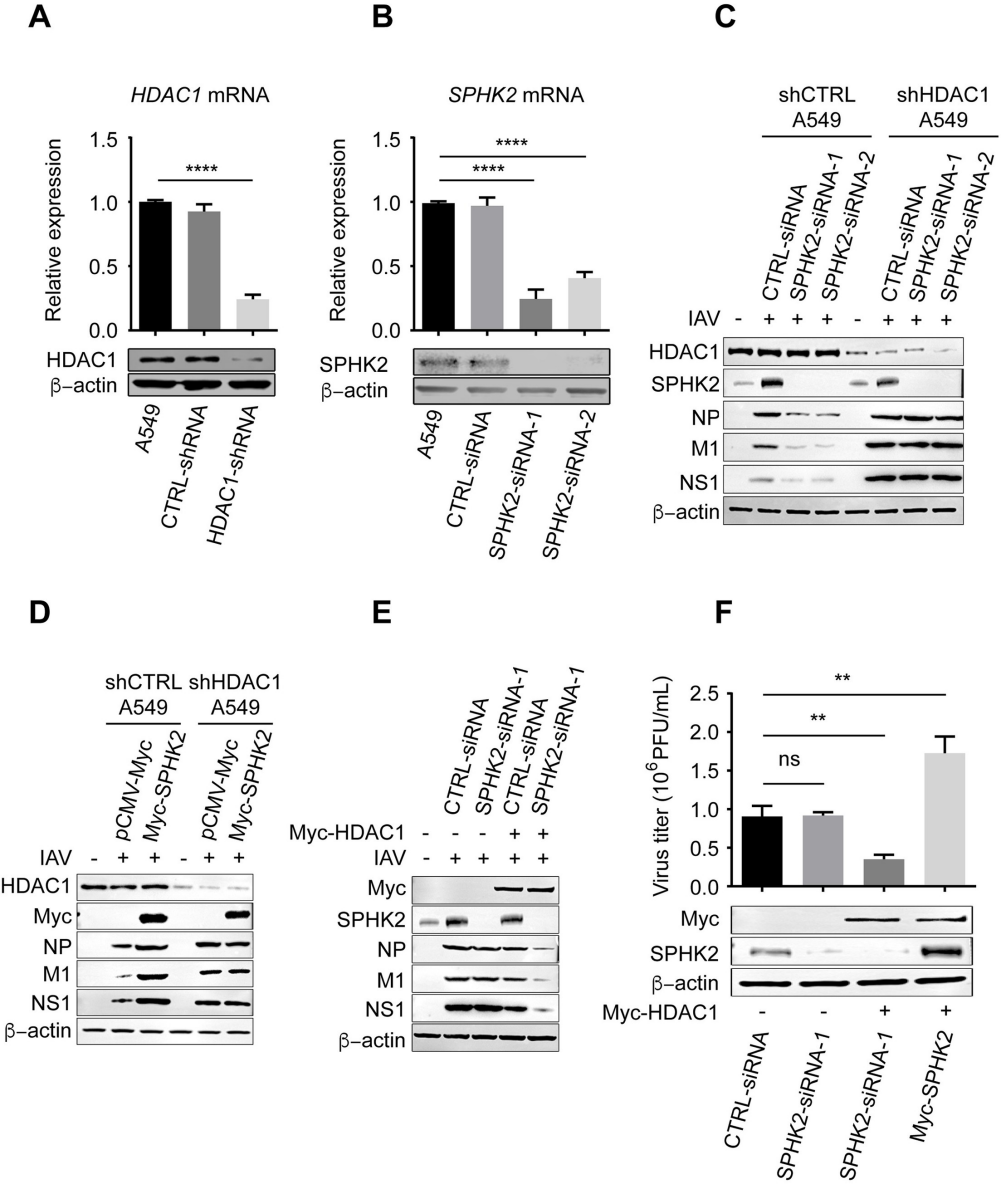
SPHK2 facilitates IAV infection through HDAC1. (**A**) HDAC1 was stably knocked down in A549 cells. The mRNA and protein levels of HDAC1 was measured by qPCR and Western blotting respectively. (**B**) A549 cells were transfected with negative control (CTRL-siRNA) or SPHK2-targeting siRNAs (SPHK2-siRNAs) to silence SPHK2, and 48 h later, the mRNA and protein levels of SPHK2 was measured by qPCR and Western blotting respectively. (**C**) shHDAC1 A549 cells were transfected with siRNAs (CTRL-siRNA or SPHK2-siRNA-1, SPHK2-siRNA-2) and infected with WSN virus at an MOI of 0.5. After 24 h infection, the expression of NP, M1 and NS1 were detected by Western blotting, β-actin was as an internal loading control. (**D**) shCTRL A549 cells or shHDAC1 A549 cells were transfected with control vector or SPHK2-expressing plasmids and infected with WSN virus at an MOI of 0.5. After 24 h infection, the expression of NP, M1 and NS1 were detected by Western blotting. (**E**) shHDAC1 A549 cells were transfected with siRNAs (CTRL-siRNA or SPHK2-siRNA-1) and HDAC1 expression was restored. After 24 h, the cells were infected with WSN virus at an MOI of 0.5 and the expression of NP, M1 and NS1 were detected by Western blotting. (**F**) HDAC1 knock-down A549 cells (shHDAC1) were treated with CTRL-siRNA, SPHK2-siRNA-1 or transfected with Myc-SPHK2 plasmids, followed by restoration of HDAC1 expression. After 24 h, the cells were infected with WSN virus at an MOI of 0.5 and the virus titers were measured by plaque assay, and Myc-tagged HDAC1, SPHK2 protein in these cells were detected by Western blotting. Data are representative of three independent experiments. ns, no significance; **, P<0.01; ****, P<0.0001.

To further understand the mode of action of how SPHK2 regulates influenza virus infection, we sought to identify certain cellular factor(s) that account for the SPHK2’s pro-viral function. It has been reported that SPHK2 associates with histone deacetylase 1 (HDAC1) in a repressor complex, and influenza A virus dysregulates HDAC1 for its successful propagation through NP protein which interacts with HDAC1 for the downregulation of its acetylation level of NP [18,39–41]. Therefore, we generated HDAC1 knock-down A549 cells (shHDAC1: shRNA targeted to HDAC1 3’UTR sequence [42]) to explore if HDAC1 is crucial for the SPHK2-mediated regulation of IAV replication. HDAC1 mRNA in shHDAC1 A549 cells was reduced approximately 80% and the protein level of HDAC1 was also detected (Fig 4A). Subsequently, shHDAC1 A549 cells were treated with siRNA targeting to SPHK2 (Fig 4B), and then infected with WSN virus. Western blotting analysis showed that compared to reduction of viral protein levels in shCTRL A549 cells, there was no discrepancy observed in viral protein synthesis between SPHK2-siRNAs treated shHDAC1 A549 cells and CTRL-siRNA treated shHDAC1 A549 cells (Fig 4C). Further, the viral protein levels of IAV (NP, M1 and NS1) in shCTRL A549 cells were increased, but there was no increase of these proteins in shHDAC1 A549 cells when overexpressed with SPHK2 (Fig 4D). Finally, we conducted gene rescue experiment [43] in HDAC1 knockdown cells and found that viral titer (Fig 4F) and viral protein levels (Fig 4E) were reduced in SPHK2-siRNA treated cells after the restoration of HDAC1 expression. Virus titer in SPHK2 overexpressing shHDAC1 A549 cells was increased after the restoration of HDAC1 expression (Fig 4F). Together, these results indicate that HDAC1 is critical for the SPHK2-mediated regulation of IAV propagation.

### The enzymatic activity of SPHK2 is dispensable for supporting IAV infection

**Fig 5:**
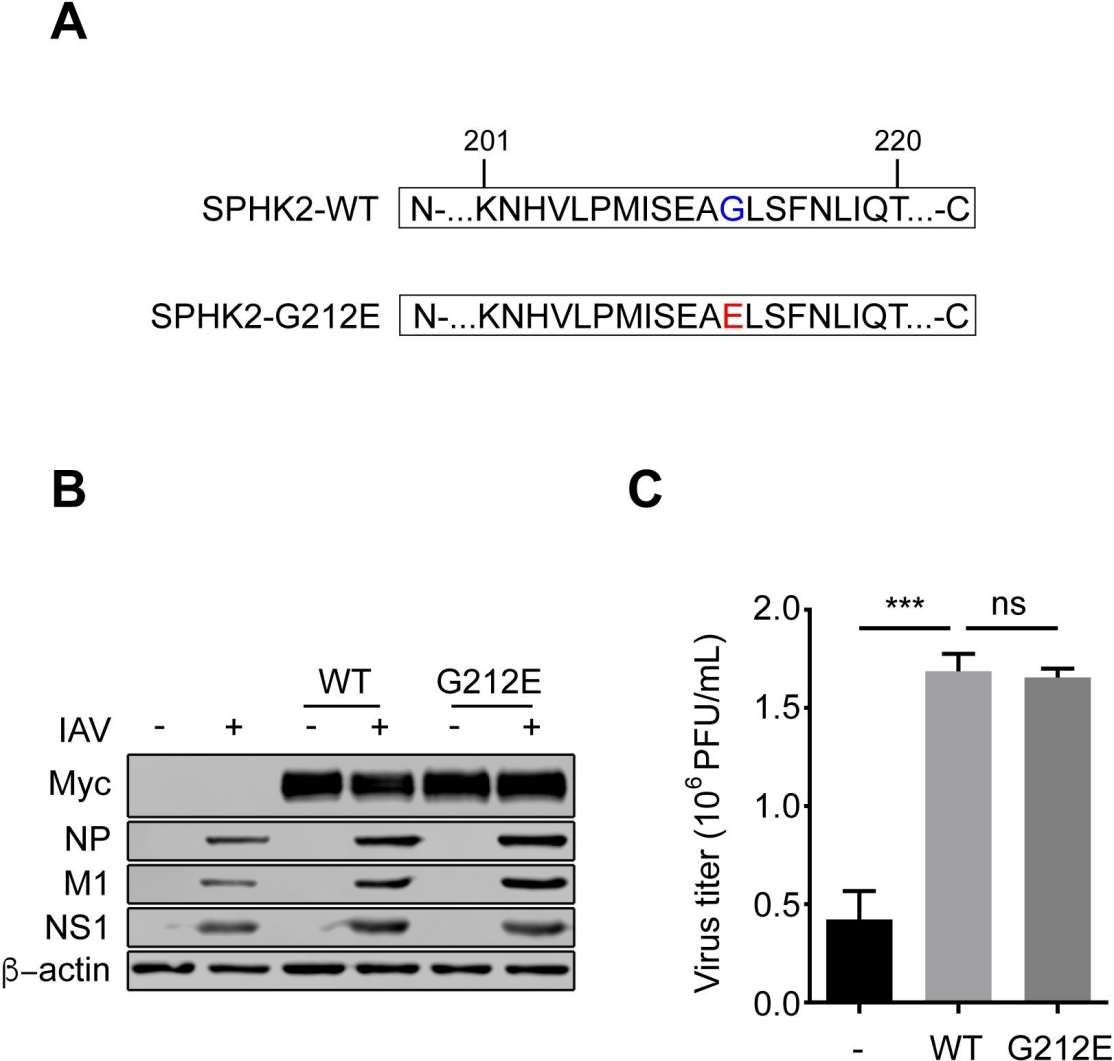
IAV replication is independent of the enzymatic activity of SPHK2. (**A**) The amino acid sequence of SPHK2 wild-type (WT) and the mutation site of SPHK2-G212E are indicated. (**B and C**) A549 cells were transfected with vectors encoding Myc-tagged SPHK2-WT or SPHK2-G212E mutants, pCMV-Myc plasmid was as a control vector (-), and infected with WSN virus at an MOI of 0.5. After 24 h, Western blotting was used to detected the NP, M1 and NS1 protein (B). After 24 h infection, cell supernatants were collected for a plaque assay to measure the virus titers in A549 cells at each condition (C). All data are representative of three independent experiments showing similar results. ns, no significance; ***, P<0.001.

Considering the enzymatic properties of SPHK2, we investigated if the enzymatic activity of SPHK2 is essential for promoting IAV infection. Consequently, a series of experiments were conducted to verify this hypothesis. It has been reported that the amino acid residue G212 is crucial for the enzymatic activity of SPHK2 [18,44]. Thus, we constructed the SPHK2-G212E mutant, cloned it into the mammalian expressing vector and verified its expression in A549 cells (Fig 5A) and that the Myc-tagged SPHK2-G212E did not affect A549 cell growth rate was also confirmed (S3A Fig). The loss of the enzymatic activity of SPHK2-G212E mutant overexpressed in A549 cells was determined (S3B Fig). Cells transfected with control vector pCMV-Myc (-), SPHK2-WT or SPHK2-G212E expressing plasmids were infected with WSN virus and cells were collected for Western blotting analysis at 24 h post infection. Unexpectedly, viral NP, M1 and NS1 protein levels had no obvious change between G212E and WT overexpressed cells (Fig 5B). Moreover, the virus titer in the supernatant of SPHK2-G212E transfected cells was nearly equal to SPHK2-WT transfected cells (Fig 5C). Therefore, we found IAV replication is independent of the enzymatic activity of SPHK2.

### SPHK2 downregulates type I IFN signaling during influenza virus infection

**Fig 6:**
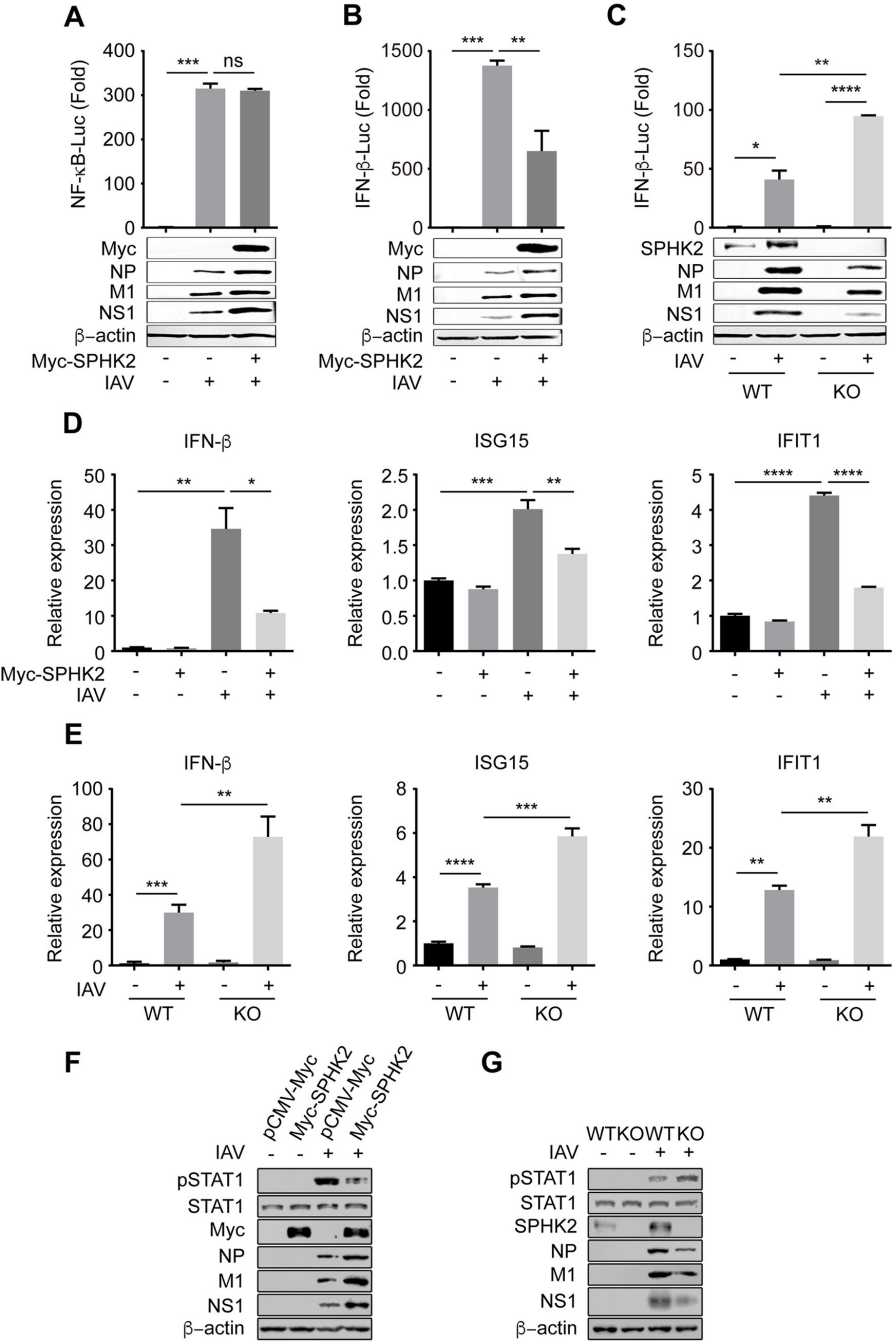
SPHK2 downregulates type I IFN signaling during IAV infection. (**A and B**) HEK293 cells were transfected with pCMV-Myc (-) or SPHK2-encoding plasmids, with luciferase plasmids (pGL3-IFNβ-promoter or pGL3-NF-κB-promoter with pRL-TK co-transfected into cells), and then infected with WSN virus at an MOI of 0.5. NF-κB (A) or IFN-β (B) activity was measured using luciferase reporter assays, and viral NP, M1, NS1 proteins in these cells were detected by Western blotting, β-actin was as an internal loading control. (**C**) A549 cells or SPHK2 knockout A549 cells were infected with WSN virus at an MOI of 0.5. IFN-β activity was measured using luciferase reporter assays, and viral NP, M1, NS1 proteins in these cells were detected by Western blotting. (**D**) A549 cells were transfected with pCMV-Myc (-) or SPHK2-expressing plasmids, and 24 h after transfection, cell were infected with WSN virus at an MOI of 0.5. The mRNA levels of IFIT1, ISG15 and IFNb was analyzed by qPCR at 24 hpi, which was normalized to the mRNA level of GAPDH. (**E**) A549 cells or SPHK2 knockout A549 cells were infected with WSN virus at an MOI of 0.5 for 24 h. The mRNA levels of IFIT1, ISG15 and IFNb was analyzed by qPCR at 24 hpi. (**F**) A549 cells were transfected with pCMV-Myc or Myc-SPHK2 plasmids, and 24 h after transfection, cells were infected with WSN virus at an MOI of 0.5. pSTAT1, STAT1, NP, M1 and NS1 protein in A549 cells were analyzed by Western blotting at 24 hpi. (**G**) A549 cells or SPHK2 knockout A549 cells were infected with WSN virus at an MOI of 0.5 for 24 h. pSTAT1, STAT1, NP, M1 and NS1 protein in A549 cells were analyzed by Western blotting at 24 hpi. Data are representative of three independent experiments. ns, no significance; *, P<0.05; **, P<0.01; ***, P<0.001; ****, P<0.0001.

Previous studies have showed that both NF-κB and type I IFNs are of great importance in preventing viral infection by boosting host innate immune responses against influenza A virus [45–47]. It has also been reported that inhibition of SPHK1, another sphingosine kinase homologous to SPHK2, suppresses virus-induced NF-κB activation [15]. Thus, we next investigated whether SPHK2 regulates the NF-κB or type I IFN signaling pathway. To this end, we first evaluated the NF-κB-promoter activity by luciferase assay in A549 cells overexpressed with SPHK2 upon IAV infection. However, we found that overexpression of SPHK2 did not have any effect on NF-κB promoter activity (Fig 6A). Overexpression or knockout of SPHK2 in A549 cells also did not affect the mRNA level of NF-κB related gene TNF-α and IL6 (S4A and S4B Fig). We next determined the effect of SPHK2 on IFN-β promoter activity during IAV infection. IAV-induced activation of IFN-β promotor was remarkably inhibited in SPHK2 overexpressing A549 cells (Fig 6B). On the contrary, knockout of endogenous SPHK2 significantly enhanced IFN-β promotor activity (Fig 6C). We also detected the mRNA levels of IFN-β and type I IFN-related genes IFIT1 and ISG15 in A549 cells transfected with control vector or plasmid encoding SPHK2. Consistently, the mRNA levels of IFN-β, IFIT1 and ISG15 were significantly reduced in the SPHK2 overexpressing cells during IAV infection (Fig 6D). Furthermore, knockout of endogenous SPHK2 enhanced the virus-triggered expression of IFN-β, IFIT1, and ISG15 at mRNA levels (Fig 6E). Western blotting analysis revealed significant decreases in pSTAT1 protein levels in SPHK2 overexpressed A549 cells (Fig 6F). Conversely, the protein levels of pSTAT1 were elevated in SPHK2 knockout A549 cells (Fig 6G). These findings reveal that SPHK2 inhibits type I IFN signaling upon influenza virus infection.

### SPHK2 interacts with TET3 and recruits HDAC1 for the regulation of IFN-β transcription

**Fig 7:**
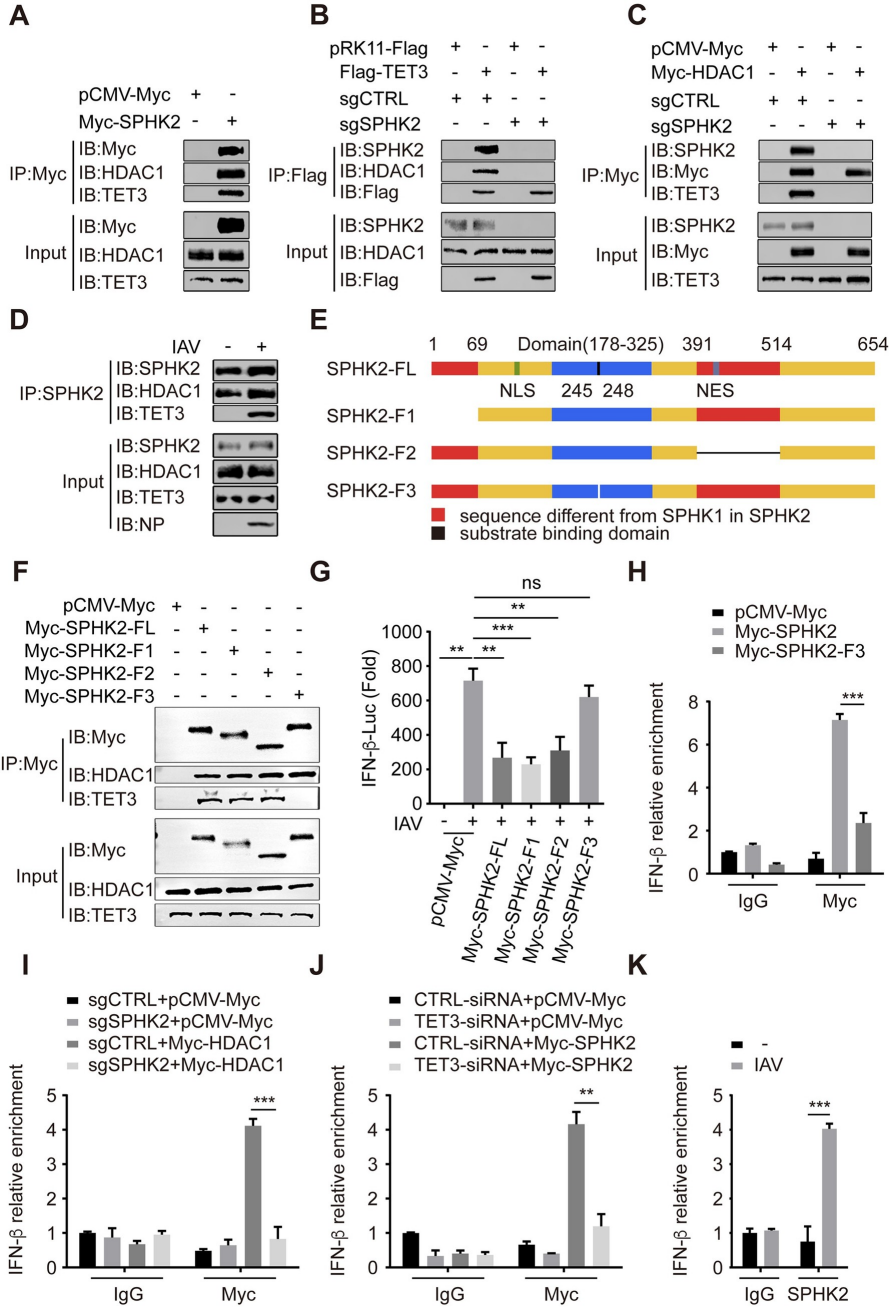
The substrate binding domain of SPHK2 interacts with TET3 and HDAC1 and inhibits IFN-β transcription. (**A**) A549 cells transfected with pCMV-Myc or Myc-SPHK2 were immunoprecipitated using anti-Myc antibody, and then Myc-tagged SPHK2, HDAC1, and TET3 proteins were analyzed by immunoblotting. (**B**) A549 cells transfected with pRK11-Flag or Flag-TET3 were immunoprecipitated using anti-Flag antibody, and then SPHK2, HDAC1, and Flag-tagged TET3 proteins were analyzed by immunoblotting. (**C**) A549 cells transfected with pCMV-Myc or Myc-HDAC1 were immunoprecipitated using anti-Myc antibody, and then SPHK2, Myc-tagged HDAC1, and TET3 proteins were analyzed by immunoblotting. (**D**) A549 cells uninfected or infected with WSN were immunoprecipitated using anti-Human SPHK2 polyclonal antibody (Proteintech, 17096-1-AP), and then SPHK2, HDAC1 and TET3 proteins were analyzed by immunoblotting. (**E**) Diagram of SPHK2-FL (full length) and deletion of SPHK2 fragments (F1, F2 and F3). (**F**) A 549 cells transfected with pCMV-Myc, Myc-SPHK2-FL, Myc-SPHK2-F1, Myc-SPHK2-F2, and Myc-SPHK2-F3 were immunoprecipitated by using anti-Myc antibody, and then Myc-tagged SPHK2, Myc-tagged SPHK2-F3, HDAC1, and TET3 proteins were analyzed by immunoblotting. (**G**) HEK293 cells were transfected with pCMV-Myc, Myc-SPHK2-FL, Myc-SPHK2-F1, Myc-SPHK2-F2, or Myc-SPHK2-F3, and then infected with WSN virus at an MOI of 0.5. IFN-β activity was measured by using luciferase reporter assays. (**H**) A549 cells transfected with pCMV-Myc (Mock), Myc-SPHK2 or Myc-SPHK2-F3, and IFN-β promoter enrichment for Myc-tagged SPHK2 or Myc-tagged SPHK2-F3 was detected by ChIP-qPCR. (**I**) IFN-β promoter enrichment for Myc-tagged HDAC1 in sgCTRL A549 cells or sgSPHK2-1 A549 cells overexpressed with Myc-tagged HDAC1 was detected by ChIP-qPCR. (**J**) A549 cells were transfected with CTRL-siRNA or TET3-siRNA, and 24 h post-transfection, cells were overexpressed with SPHK2. IFN-β promoter enrichment for Myc-tagged SPHK2 was detected by ChIP-qPCR. (**K**) A549 cells were uninfected or infected with WSN, and IFN-β promoter enrichment for SPHK2 was determined by ChIP-qPCR. Data are representative of three independent experiments. ns, no significance; **, P<0.01; ***, P<0.001.

After a further confirmation that IFN-β promotor activity was regulated by HDAC1 via SPHK2, but not by the enzymatic activity of SPHK2 (S5 Fig), we raised a question that if there is any key protein participating in the SPHK2-mediated regulation of IFN-β promoter. The DNA demethylase TET3 has been previously described to promote the interaction of HDAC1 with the IFN-β promoter and inhibits type I IFN production [38]. Additionally, SPHK2 and HDAC1 has been proved to bind to each other in a corepressor complex [18]. Therefore, we hypothesized that SPHK2 may function as an adapter protein between TET3 and HDAC1. Because of the binding capacity of TET3 to IFN-β promoter, HDAC1 which binds to SPHK2 may be recruited to IFN-β promoter to inhibit IFN-β transcription. To test this, we examined whether SPHK2 interacts with TET3. In SPHK2 overexpressing cells, both endogenous TET3 and HDAC1 were precipitated by Myc-tagged SPHK2 (Fig 7A). Converse to the situation in sgCTRL A549 cells, overexpressed Flag-TET3 could not precipitate HDAC1 in sgSPHK2-1 A549 cells (Fig 7B). Myc-tagged HDAC1 also could not precipitate endogenous TET3 in sgSPHK2-1 A549 cells (Fig 7C). Moreover, endogenous SPHK2 could precipitate HDAC1 and TET3 in IAV infected A549 cells, but could not precipitate TET3 in uninfected A549 cells (Fig 7D). Next, colocalization of SPHK2 with HDAC1 and TET3 in uninfected and infected A549 cells were observed by immunofluorescence analysis. SPHK2 colocalized with HDAC1 in uninfected A549 cells transfected with Myc-HDAC1, but not with TET3 in uninfected A549 cells transfected with Flag-TET3. Upon IAV infection, both Myc-tagged HDAC1 and Flag-tagged TET3 colocalized with SPHK2 (S6A Fig). According to the above results, we wonder to know whether the entry of SPHK2 into the nucleus is caused by HDAC1 or IAV infection. The immunofluorescence analysis showed that SPHK2 proteins still translocated into the nucleus of shHDAC1 A549 cells after viral infection (S6B Fig), which suggests that it was the IAV infection that caused SPHK2 to enter into the nucleus. Both SPHK2 and SPHK1 are pro-viral factors. However, SPHK1 regulates NF-κB pathway [15], while we found SPHK2 regulated type I IFN pathway. To get a more detailed understanding of the underlying mechanisms, we next constructed Myc-SPHK2-F1 and Myc-SPHK2-F2 truncated plasmids, according to the structure alignment between SPHK1 and SPHK2, and Myc-SPHK2-F3 plasmids with the substrate binding domain mutation to find out which domain regulates IFN-β transcription (Fig 7E). We found that Myc-SPHK2-F3 failed to precipitate TET3, while Myc-SPHK2-FL, Myc-SPHK2-F1 and Myc-SPHK2-F2 could precipitate TET3 (Fig 7F). Immunoblotting analysis with endogenous SPHK2 antibody in Myc-antibody precipitated complexes from Myc-tagged full length of SPHK2 or Myc-tagged SPHK2 truncated fragments (Myc-SPHK2-F1, Myc-SPHK2-F2, Myc-SPHK2-F3) overexpressed A549 cells demonstrated that the above result is not due to the structural damage to the protein, but because of the loss of specific functions in these deletions (S7A Fig). Subsequently, we found that Myc-SPHK2-F3 also failed to reduce the promoter activity in IAV infected A549 cells (Fig 7G). However, we also found that the overexpressed mutant SPHK2 (G212E) that has lost enzymatic activity could precipitate HDAC1 and TET3 (S7B Fig). Finally, we conducted ChIP assays to reveal how SPHK2, TET3 and HDAC1 cooperate and interact with IFN-β promoter. ChIP-qPCR analysis with antibody against Myc-tag showed that SPHK2-FL but not SPHK2-F3 was recruited to IFN-β promoter in A549 cells (Fig 7H). IFN-β promoter enrichment was elevated in A549 cells transfected with Myc-SPHK2-G212E, Myc-SPHK2-F1 and Myc-SPHK2-F2 (S7C Fig). In addition, IFN-β promoter in sgSPHK2-1 A549 cells could not be enriched by Myc-tagged HDAC1 compared with that in sgCTRL A549 cells (Fig 7I). To address the importance of TET3 protein in SPHK2’s binding to IFN-β promoter, we transfected TET3-siRNA or CTRL-siRNA to A549 cells. We observed that IFN-β promoter enrichment was disrupted in Myc-tagged SPHK2 overexpressed A549 cells transfected with TET3-siRNA (Fig 7J). And we also found that IFN-β promoter enrichment was increased in IAV infected A549 cells (Fig 7K). In order to evaluate the specificity of SPHK2 in the regulation of IFN-β promoter, we selected two promoters (TGF-β1 [36] and IL6 [35]) regulated by TET3 for ChIP-qPCR analysis. We found that both TGF-β1 and IL6 promoter could not be enriched by Myc-tagged SPHK2 and endogenous SPHK2 in IAV infected A549 cell (S8 Fig). Collectively, these data demonstrate that the substrate binding domain of SPHK2 is responsible for the interaction with TET3, and then recruits HDAC1 to IFN-β promoter and specifically regulates IFN-β transcription through HDAC1.

### SPHK2 negatively regulates IFN-β transcription by enhancing deacetylation of IFN-β promoter through TET3 and HDAC1

**Fig 8:**
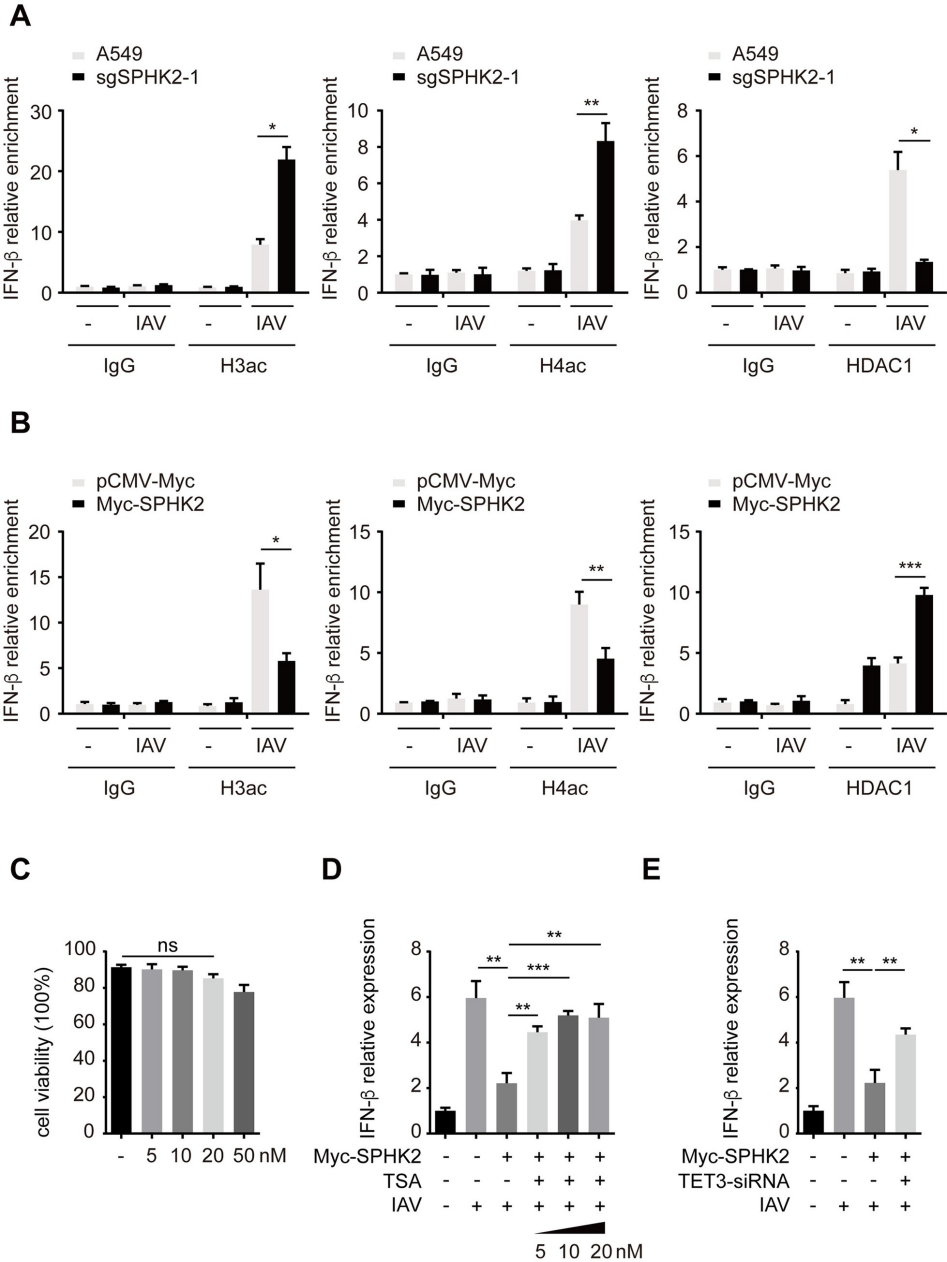
SPHK2 inhibits acetylation of IFN-β promoter through TET3 and HDAC1. (**A**) A549 cells and sgSPHK2-1 A549 cells were infected with IAV (WSN), MOI = 0.5, 24 h post infection, IFN-β promoter enrichment for H3ac, H4ac and HDAC1 antibodies was detected by ChIP-qPCR. (**B**) A549 cells transfected with pCMV-Myc or Myc-SPHK2 were infected with IAV (WSN), MOI = 0.5, 24 h post infection, IFN-β promoter enrichment for H3ac, H4ac and HDAC1 antibodies was detected by ChIP-qPCR. (**C**) A549 cells were treated with solvent (-) or TSA (5, 10, 20 or 50 nM) for 72 h, cell viability was detected by Cell Counting Kit-8. (**D**) A549 cells were treated with TSA (5, 10 and 20 nM), then transfected with Myc-SPHK2 and infected with WSN, MOI = 0.5, after 24 h, IFN-β mRNA level was measured by qPCR, and normalized to GAPDH. **(E**) A549 cells were transfected with TET3-siRNA, then transfected with Myc-SPHK2 and infected with WSN, MOI = 0.5, after 24 h, cells IFN-β mRNA level was measured by qPCR. Data are representative of three independent experiments. ns, no significance; *, P<0.05; **, P<0.01; ***, P<0.001.

Given that SPHK2 associated with TET3 and HDAC1 to inhibit IFN-β transcription, we thus further determined whether the deacetylation function of HDAC1 in the regulation of SPHK2 on IFN-β promoter was necessary for the mechanism. ChIP assay showed that compared to IFN-β promoter in IAV infected A549 cells, it was highly enriched by acetyl-histone H3 and H4 antibodies in IAV infected sgSPHK2-1 A549 cells. Consistently, ChIP with HDAC1 antibody enriched much more IFN-β promoter in A549 cells upon IAV infection, and the enrichment was markedly decreased after the knockout of SPHK2 (Fig 8A). ChIP assay with acetyl-histone H3 and H4 antibodies also showed that compared to pCMV-Myc vector transfected A549 cells upon IAV infection, the enrichment of IFN-β promoter was obviously decreased when transfected with Myc-SPHK2. Consistently, IFN-β promoter in IAV infected A549 cells that overexpressed with SPHK2 were much more enriched by HDAC1 antibodies, than cells transfected with pCMV-Myc (Fig 8B). Next, to validate the importance of the deacetylation function of HDAC1 in the regulation of SPHK2 on IFN-β promoter, HDAC1’s deacetylation activity in cells was inhibited with HDAC1 inhibitor Trichostatin A (TSA). Cell viability assay showed that TSA (5, 10 and 20 nM) had no cytotoxicity to A549 cells in the experimental conditions (Fig 8C). The qPCR analysis further validated that during IAV infection, IFN-β mRNA level was elevated in SPHK2 overexpressed A549 cells after TSA treatment (Fig 8D). When HDAC1’s deacetylation activity was inhibited in cells treated with TSA, overexpression of SPHK2 in these cells could not inhibit the mRNA levels of IFN-β. Moreover, IFN-β mRNA level was also elevated in IAV infected A549 cells overexpressed with SPHK2 after the knockdown of TET3 (Fig 8E). This finding indicates that the deacetylation function of HDAC1 is essential for the SPHK2-mediated mechanisms. In IAV infected cells, SPHK2 interacts with HDAC1 and TET3, and HDAC1 was recruited to IFN-β promoter by the interaction, subsequently histone 3 and histone 4 in IFN-β promoter region were deacetylated, leading to the inhibition of IFN-β transcription.

## Discussion

**Fig 9:**
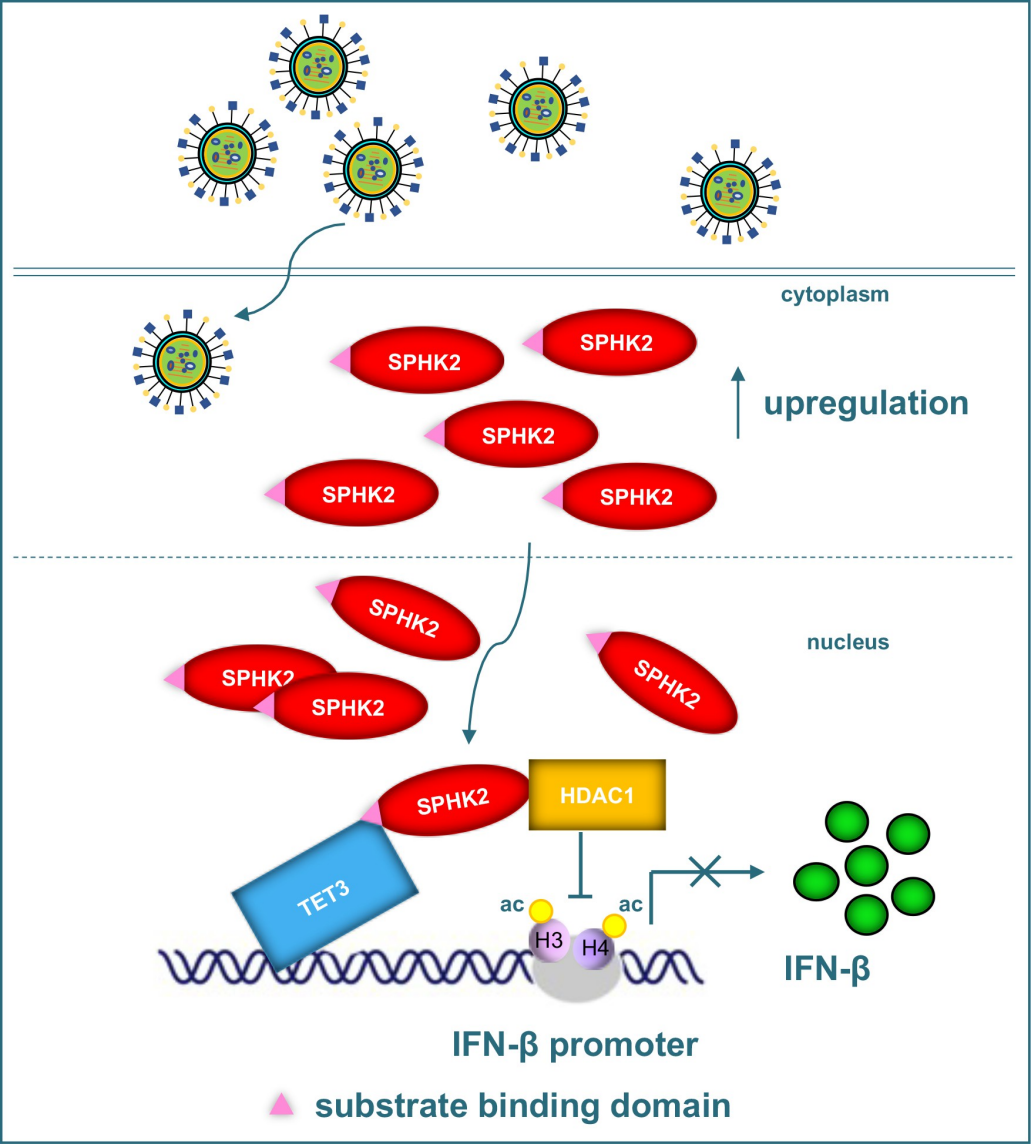
SPHK2 interacts with HDAC1 and TET3 to inhibit IFN-β transcription. The expression of SPHK2 in IAV infected cells is upregulated and then the location of SPHK2 is shifted from cytoplasm to nucleus. Afterwards, SPHK2 accumulated in the nucleus interacts with HDAC1 and its substrate binding domain also interacts with TET3 that bands to IFN-β promoter. Therefore, HDAC1 is recruited to IFN-β promoter and inhibited IFN-β transcription by enhancing the deacetylation of IFN-β promoter.

In this study, we demonstrate that SPHK2, an IAV-induced and pro-viral host factor, depends on the histone deacetylase HDAC1, negatively regulates type I IFN response, and thus facilitates IAV infection. Unexpectedly, the core enzymatic activity of SPHK2 that inhibits HDAC1 by producing S1P is unrelated to the increased IAV propagation. More interestingly, SPHK2 using the substrate binding domain to interact with TET3, an inhibitor of IFN-β production with DNA binding activity, is recruited to the IFN-β promoter. Therefore, the IFN-β transcription is inhibited by the SPHK2-interacting cooperator HDAC1, and IAV replication was increased within host cells (Fig 9).

We found that SPHK2 was obviously upregulated in IAV infected A549 cells, and its location was shifted from cytoplasm to nucleus, suggesting SPHK2 may play a role in influenza virus infection. SPHK1, homologous to SPHK2, has been reported to possess a pro-viral property. Inhibition of SPHK1 suppresses virus-induced NF-κB activation [15,48]. Therefore, SPHK2 may also have a similar character with SPHK1 during IAV infection. However, different from SPHK1 which upregulates NF-κB activity, we found that SPHK2 downregulates type I IFN responses during IAV infection. SPHK2, essential for sphingolipid metabolism, catalyzes the phosphorylation of sphingosine which is derived from the N-deacetylation of ceramide by ceramidase, brings it into generating S1P, and activates the S1P receptor signaling [49]. However, we found that the catalytic-incompetent SPHK2 mutant has the same ability to support IAV replication with the WT SPHK2 (Fig 5). Thus, we believe that the pro-viral function of SPHK2 is independent of its enzymatic activity. It has been reported that endogenous lipid peroxidation restricts the replication of some HCV strains, leading to the increased viral persistence [23], SPHK2 maintains KSHV latency in virus infected endothelial cells and promotes CHIKV replication *in vitro* [20,22]. Moreover, the sphingosine precursor ceramide inhibits IAV infection *in vitro* [50], S1P receptor signaling protects mice from pathogenic influenza [51], while S1P lyase (SPL) enhances type I IFN production to restrict IAV replication [52,53], indicating that sphingolipid metabolism occupies an important place in influenza virus infection. However, as one member of sphingolipid metabolism, SPHK2 facilitates IAV infection independent of its enzymatic activity, suggesting the sphingolipid metabolism function is not involved in SPHK2 enhanced IAV replication but by another novel mechanism. Besides, genetic deletion of SPHK2 impairs dengue virus (DENV) infection, but inhibition of SPHK2 activity does not affect DENV replication, which also suggests SPHK2 activity may not be required for some viruses such as DENV and IAV [54]. Therefore, we wonder to know whether the SPHK2 protein itself or there might be some unknown proteins involved in the regulation and promotion of IAV infection.

HDACs play multiple roles during viral infection. For instance, HDAC11 and HDAC4 are involved in the modulation of HBV replication [55], HDAC8 is required for influenza A virus entry [56], HDAC6 is crucial for IAV-induced apoptosis of infected cells [57], HDAC2 is a key factor of host anti-influenza virus response [58]. Endogenous SPHK2 associates with HDAC1 and interacts with histone H3. SPHK2-generated S1P specially binds to HDAC1 to inhibit their enzymatic activities, enhancing p21 and c-fos transcription [18]. It has been reported that HDAC1 inhibits IAV infection [41], but intriguingly, others have also reported that HDAC1 facilitates IAV infection by supporting the nuclear preservation of NP and negatively regulating TBK1-IRF3 pathway [39]. Thus, it is attractive to determine whether HDAC1 participates in SPHK2 facilitated IAV infection.

As previously reported, TET3 exerts a methylation epigenetic regulation of the corresponding promoters under various physiological conditions [35–38]. Specially, TET3 can function in a DNA demethylation-independent manner and binds to the IFN-β promoter and leads to the recruitment of HDAC1 to the IFN-β promoter, consequently inhibiting IFN-β transcription under viral infection [38]. However, no evidence has been found to support a direct interaction between TET3 and HDAC1. Of note, our study has proved that SPHK2 is a negative regulator of IFN-β transcription and HDAC1 is known to be a binding partner of SPHK2, not SPHK1 [18], thus we speculate that TET3 binds to HDAC1 through SPHK2, leading to the recruitment of HDAC1 and the inhibition of IFN-β transcription. This assumption has been verified in our study (Figs 7 and S9). We have found SPHK2 serves as an adaptor protein between TET3 and HDAC1. Of note, in Fig 7D, endogenous SPHK2 could precipitate HDAC1 and TET3 in IAV infected A549 cells, but could not precipitate TET3 in mock infected cells. Our explanation is that, compared to uninfected A549 cells, IAV infection induces a large amount of accumulation of SPHK2 in the nucleus, and perhaps some modifications on SPHK2 proteins, leading to the interaction with HDAC1 and TET3. This may be a way of protecting cells from non-physiological activation of IFN-β. According to the previous reports and the results of Fig 2A, we speculate that the necessary and small amount of SPHK2 present in nucleus could interact with HDAC1 to regulate other cellular progress in normal conditions [18]. Upon IAV infection, some modifications or changes were created in both SPHK2 and TET3, leading to SPHK2’s accumulation in the nucleus and the interaction of SPHK2-HDAC1 complex with TET3, subsequently the specific binding of the IFN-β promoter recruited by TET3 in response to viral infection. However, it’s still confusing that this process is unrelated with the enzymatic activity of SPHK2, but depends on its substrate binding domain, suggesting a mechanism distinct from SPHK2 and HDAC1 regulated p21 and c-fos transcription. Finally, we have found TET3 binds to the substrate binding domain of SPHK2, which inhibits the enzymatic activity of SPHK2 and releases the deacetylation function of HDAC1 to the IFN-β promoter, resulting in IFN-β transcriptional inhibition. Furthermore, the result is in accordance with our previously findings that inhibition of SPHK2 in IAV infected mice by ABC294640 which competitively binds to the substrate binding domain of SPHK2 increases IFN-β production, thus protecting mice from lethal influenza A virus infection [14].

In summary, our study determines that SPHK2 displays a pro-IAV function which is dependent on HDAC1, and the regulation of IAV replication is independent of the enzymatic activity of SPHK2. Furthermore, SPHK2 negatively regulates type I IFN signaling during IAV infection. Most importantly, SPHK2 interacts with TET3 through its substrate binding domain and recruits HDAC1 to the IFN-β promoter to inhibit IFN-β transcription by increasing the deacetylation of IFN-β promoter. Since the two TET3 regulated TGF-β1 and IL6 promoters could not be enriched by SPHK2 in IAV infected cells, we speculate that the SPHK2-mediated mechanism which specially modulate the IFN-β promoter is dependent both on the binding properties of TET3 and SPHK2 induced by IAV infection. However, the mechanism of how IAV induces the accumulation of SPHK2 to the nucleus is not extensively elucidated. There are reports that protein kinase D mediated phosphorylation regulates the nuclear export of SPHK2 [59], and serum deprivation can also induce SPHK2’s translocation in the cells [24]. Our findings demonstrate that the accumulation of SPHK2 in the nucleus was specific to influenza A virus infection, indicating that there may be existing other unknow factors related to IAV infection that make the difference on the cellular location of SPHK2 in response to IAV infection, and the mechanism needs to be further investigated in the future work. Anyway, our findings further broaden our horizon regarding the multiple immune regulation functions of SPHK2 and the special role of SPHK2 in promotion of IAV infection. The new understanding of the interplay between IAV and SPHKs may promote the development of alternative or next generation of anti-IAV strategies.

## Materials and methods

### Cells and viruses

Human embryonic kidney 293 (ATCC), Madin-Darby canine kidney (MDCK) epithelial cells (ATCC), human lung epithelial lung cell line A549 (ATCC), and the related knock-down A549 cell lines were grown at 37°C and 5% CO_2_ in Dulbecco’s modified Eagle’s medium (DMEM) supplemented with high glucose, GlutaMax, pyruvate (Gibco), 10% fetal bovine serum (FBS, Lonsera), and 1% P/S (100 units/ml penicillin, 100 μg/ml streptomycin; Gibco). Influenza virus A/PR/8/34 H1N1 (a gift from Dr. Wenbao Qi at the Key Laboratory of Veterinary Vaccine Innovation of the Ministry of Agriculture) and A/WSN/34 H1N1 (a gift from Dr. Wenjun Song at Guangzhou Medical University) strains were propagated and titrated on MDCK cells, and then stored at -80°C. EV71 (Xiangyang-Hubei-09) and ZIKV (z16006) strains were kindly provided by Dr. Zhen Luo at Jinan University. For viral infection, the cell monolayers were washed twice with PBS, the virus inoculum was diluted in infection media, i.e., serum-free DMEM containing 0.3% BSA (Sigma-Aldrich), 1% P/S and 1 μg/ml TPCK-trypsin (Sigma-Aldrich). Next, cells were incubated with virus at 37°C for 1 h. After removing the virus inoculum, cells were washed twice with PBS, subsequently replaced with infection media. Generally, cells were infected at the multiplicity of infection (MOI) of 0.01–5.0 plaque-forming units (PFUs) per cell.

### Regents and antibodies

HDAC inhibitor Trichostatin A (TSA) was purchased from Sigma (T1952). SPHK1 inhibitor SK1-I was purchased from MCE (HY-119016). Trypan blue solution was purchased from Gibco (15250061). NE-PER Nuclear and Cytoplasmic Extraction Reagents was purchased from Thermo Fisher (78833). NBD sphingosine was purchased from Avanti (810205). ATP was purchased from Invitrogen (PV3227). Antibodies against influenza A virus NP, human GAPDH, Lamin B1 and β-Actin were purchased from Abcam (ab128193, ab181602, ab16048, ab6276). Anti-human Myc and Flag tagged antibodies were purchased from Sigma (M4439, F1804). The antibodies against human HDAC1, STAT1 and pSTAT1 were obtained from CST (34589, 9172S, 9167S). The antibodies against influenza A virus M1, NS1 and Enterovirus 71 VP1 antibody were obtained from GeneTex (GTX125928, GTX25990, GTX132338). Anti-human TET3 antibody was purchased from Affinity (DF13335). Anti-human SPHK2 polyclonal antibody was purchased from Proteintech (17096-1-AP). Anti-human SPHK2 monoclonal antibody and Zika virus NS1 monoclonal antibody was purchased from Invitrogen (9C5E1, EA88). FITC-Goat polyclonal to influenza A virus (ab20388), Goat Anti-Rabbit IgG H&L (Alexa Flour 647, ab150079) and Goat Anti-Mouse IgG H&L (Alexa Flour 555, ab150114, Alexa Flour 568, ab175473) were purchased from Abcam. DAPI (C1002) was purchased from Beyotime.

### Plasmids design and construction

The constructs generated in our study are presented in Table 1. The pGL3-IFN-β-promoter and pRL-TK plasmids were obtained as previously described [60]. The site-directed mutagenesis of SPHK2 (G212E) was generated using the mutation primers by Fast Mutagenesis system (Transgen) according to the manufacturer’s protocol. The mutation primers are as follows: G212E-F: 5’-TGATCTCTGAAGCTGGGCTGTCCTTC-3’, G212E-R: 5’-CCAGCTTCAGAGATCATGGGAAGCA-3’.

**Table 1:** Plasmid constructs used in the study.

Plasmids	Description	Reference /source
pCMV-Myc	The empty vector with Myc tag	[71]
pRK11-Flag	The empty vector with Flag tag	[71]
pCMV-Myc-SPHK2	pCMV-Myc containing human SPHK2 full-length coding region	This study
pCMV-Myc-SPHK2-G212E	pCMV-Myc containing human SPHK2 sequence with G212E mutation	This study
pCMV-Myc-SPHK2-F1	pCMV-Myc containing human SPHK2 sequence with 1–69 aa deletion	This study
pCMV-Myc-SPHK2-F2	pCMV-Myc containing human SPHK2 sequence with 391–514 aa deletion	This study
pCMV-Myc-SPHK2-F3	pCMV-Myc containing human SPHK2 sequence with 245–248 aa deletion	This study
pCMV-Myc-HDAC1	pCMV-Myc containing human HDAC1 full-length coding region	This study
pRK-Flag-TET3	pRK11-Flag containing TET3 full-length sequence	This study

### Generation of CRISPR-Cas9 knock out cell lines and RNA-i transduced stable cell lines

SPHK2 knock out A549 cells were constructed using the sgRNA lentivirus packaging system as previously described [61]. HDAC1 knock down A549 cells were constructed using the shRNA lentivirus packaging system as previously described [42]. The KO efficiency of SPHK2 or the knock down efficiency of HDAC1 in A549 cells was confirmed by qPCR and Western blotting assay. The sequences of SPHK2 sgRNA and HDAC1 shRNA used in this study are as follows: sgSPHK2-1-F: 5’-CACCGTTAAGCCATAGTAGGCTGGT-3’, sgSPHK2-1-R: 5’-AAACACCAGCCTACTATGGCTTAAC-3’; sgSPHK2-2-F: 5’-CACCGCTGTTACATCAACGGGACCC-3’, sgSPHK2-2-R: 5’-AAACGGGTCCCGTTGATGTAACAGC-3’; sgSPHK2-3-F: 5’-GGTCTTAAGCCATAGTAGGC-3’, sgSPHK2-3-R: 5’-GCCTACTATGGCTTAAGACC-3’. The 3’-UTR targeting sequence for shHDAC1 was: 5’-CGTTCTTAACTTTGAACCAATA-3’ [42].

### Interfering RNA design and transfection

siRNA and control siRNA were designed and synthesized by Sangon Biotech. For silencing, A549 cells were seeded in 12 well plates (2×10^5^ cells/well), at 80–90% confluence, cells were transfected with the indicated siRNA at 100 nM with 3μL RNAiMax reagent (Invitrogen) according to the indicated protocol. The sequences of siRNA used in this study are as follows: SPHK2-siRNA-1-F: 5’-GGGAGGAAGCUGUGAAGAUTT-3’, SPHK2-siRNA-1-R: 5’-AUCUUCACAGCUUCCUCCCTT-3’; SPHK2-siRNA-2-F: 5’-AGACAGAACGACAGAACCATT-3’, SPHK2-siRNA-2-R: 5’-UGGUUCUGUCGUUCUGUCUTT-3’; TET3-siRNA: 5’-AGGCCAAGCUCUACGGGAA-3’ [62].

For the overexpression of SPHK2, SPHK2-G212E, HDAC1 or other fragments, the Myc-SPHK2, Myc-SPHK2-F1(Δ1–69), Myc-SPHK2-F2(Δ391–514), Myc-SPHK2-F3(Δ245–248), Myc-SPHK2-G212E or Myc-HDAC1 expression vector was transfected into A549 or 293T cells. The plasmid DNA was transfected with LipoD293 (Signagen) as recommended protocol provided by the manufacturer.

### Cell viability assay

A549 cells were seeded in 96 well plates (1×10^4^ cells/well), at 90% confluence, cells were then treated with TSA at concentration of 5, 10, 20 or 50 nM (experiment groups), wells just only supplemented with complete medium were blank groups, and cells with no treatment were control groups. After 72 h, supernatants were discarded, fresh complete medium and CCK8 reagent (Beyotime, C0038) was added to the groups. After shaking, 96 well plates were incubated at 37°C for 25 to 60 min. Finally, the absorbances were measured at 450 nm on automatic microplate reader (Biotek).

### Cell growth rate analysis

A549 cells or sgSPHK2-1 A549 cells were seeded in 12 well plates (1×10^5^ cells/well). A549 cells were overexpressed with Myc-tagged SPHK2 or SPHK2-G212E by using reverse transfection. In next 3 days, cells were digested and counted every 24 h by using trypan blue on blood counting chamber. The growth curves of cells with different treatment were obtained by GraphPad Prism 6 software.

### Fluorescence-based SPHK2 activity assay

A549 cells or sgSPHK2-1 A549 cells were seeded in 6 well plates (5×10^5^ cells/well). At 80–90% confluence, A549 cells were then transfected with pCMV-Myc, Myc-SPHK2 or Myc-G212E plasmid. After 24 h, cells were collected and transferred to lysis buffer (20 mM Tris, pH 7.4, 20% glycerol, 1 mM 2-mercaptoethanol, 1 mM EDTA, 5 mM sodium orthovanadate, 40 mM glycerophosphate, 15 mM NaF, 10 μg/ml leupeptin, aprotinin, and soybean trypsin inhibitor, 1 mM phenylmethylsulfonyl fluoride, and 0.5 mM 4-deoxypyridoxine), then repeated the freeze-thawing cycle in this lysis buffer for 3 times (10 min/time) [63]. Cell lysates were centrifuged at 14,000 ×g for 30 min to remove cellular debris. Supernatants were collected for subsequent SPHK2 activity assay. Supernatants (10–15 μL) was incubated with NBD-sphingosine (dissolved in 5% Triton X-100) and ATP in SPHK2 reaction buffer (30 mM Tris-HCl, pH 7.4, 0.05% Triton X-100, 200 mM KCl, and 10% glycerol), 10 mM NaF and semicarbazide were added to prevent potential degradation of formed NBD-S1P. After incubation for 30 min at 30°C, 100 μL 1 M potassium phosphate buffer, pH 8.5, was added followed by 500 μL CHCl3/MeOH 2:1 [64]. After several vortex, phases were separate by centrifugation at 15,000 ×g for 30 s. An aliquot of the upper aqueous layer was transferred into 96-well plate, following by 100 μL of dimethylformamide. Fluorescence intensity was measured in automatic microplate reader (BioTek) with excitation at 485 nm and emission at 538 nm. The reaction mixture containing no SPHK2 served as blank, The SPHK2 activity in samples are normalized to control groups.

### Cytoplasmic and nuclear protein extraction

10×10^6^ cells were transferred to 1.5 mL microcentrifuge tube and centrifuged at 500 ×g for 3 min, then the supernatant was discarded. 200 μL ice-cold Cytoplasmic Extraction Reagent I (CER I) was added to the cell pellet. Afterwards, the tube was vortexed vigorously for 15 seconds and incubated on ice for 10 min. 11 μL ice-cold Cytoplasmic Extraction Reagent II (CER II) was added to the tube. The tube was vortexed vigorously for 5 seconds and incubated on ice for 1 min. The tube was vortexed vigorously for 5 seconds again and centrifuged at 16,000 ×g for 5 min. The supernatant obtained from the centrifugation was cytoplasmic extract. After transferring the supernatant, 100 μL ice-cold Nuclear Extraction Reagent (NER) was added to the cell pellet. The tube was vortexed vigorously for 15 seconds and incubated on ice for 40 min, a continue vortex for 15 seconds every 10 min was needed during the incubation. The tube was centrifuged at 16,000 ×g for 10 min. The supernatant obtained from the centrifugation was nuclear extract. The extracts were stored at -80°C until use.

### Co-immunoprecipitation

Cells were transfected with Myc-SPHK2, Flag-TET3 or Myc-HDAC1. After 24 h transfection, the cells were washed and then lysed for 30 min at ice with Triton X-100 lysis buffer (20mM Tris-HCl pH = 7.5, 100 mM NaCl, 0.5 mM EDTA, 5% glycerol, 1% Triton X-100) containing a protease inhibitor cocktail (Beyotime). After a vortex at 4°C for 1 h, lysed cells were centrifuged, the supernatants were obtained and incubated with the indicated antibodies (anti-Myc (M, M4439), anti-Flag (M, F1804) antibody from Sigma or anti-human SPHK2 (R, 17096-1-AP) antibody from Proteintech) and protein A/G affinity beads (Beyotime) at 4°C for 4 h with gentle shaking. Subsequently, the beads were centrifuged and washed three times with ice-cold PBS, boiled in 6×SDS loading buffer for 8 min, and analyzed by Western blotting with the indicated antibodies (anti-Myc, anti-SPHK2 (R, 17096-1-AP), anti-HDAC1 (R, 34589), anti-Flag or anti-TET3 (R, DF13335) antibody).

### Western blotting analysis

Cells were lysed in TritonX-100 lysis buffer with protease inhibitor cocktail (100×; Thermo Fisher) for 30 min on ice and then centrifuged at 4°C, 16,000 ×g for 10 min. The supernatants with 6×SDS loading buffer were boiled for 8 min and stored at -80°C. The samples were separated by SDS-PAGE and transferred onto a PVDF membrane. The membrane was blocked and shook with 5% non-fat milk (Biorad) in 1×TBST for 1 h at room temperature. Then the membrane was incubated with the indicated primary antibodies followed by HRP-conjuncted secondary antibodies. The expression of protein was detected by Odyssey Fc (Li-COR), and data were analyzed by Image Studio V5.2 (Li-COR).

### RNA Extraction and real-time q-PCR

Total RNA was obtained from the cultured cells with RNA fast 200 kit (Fastagen Biotech, Shanghai, China) according to the indicated instructions. cDNA was synthesized with commercial PrimeScript RT Master Mix (Takara, Tokyo, Japan). The quantitative real-time PCR (qPCR) reaction was performed on a LightCycler 96 System (Roche, Hong Kong, China). The data represent the mRNA expression levels of experimental genes normalized to the reference gene GAPDH. Relative expression values were calculated by using the 2ˆ^-ΔΔCt^ method [65,66]. The primers used in the qPCR are as follows: qGAPDH-F: 5’-AGCCTCAAGATCATCAGCAATGCC, qGAPDH-R: 5’-TGTGGTCATGAGTCCTTCCACGAT-3’; qIFIT1-F: 5’-AGAAGCAGGCAATCACAGAAAA-3’, qIFIT1-R: 5’-CTGAAACCGACCATAGTGGAAAT-3’; qISG15-F: 5’-CGCAGATCACCCAGAAGATCG-3’, qISG15-R: 5’-TTCGTCGCATTTGTCCACCA-3’; qIFNb-F: 5’-CGCCGCATTGACCATCTA-3’, qIFNb-R: 5’-GACATTAGCCAGGAGGTTCTC-3’; qSPHK2-F: 5’-ATGGACACCTTGAAGCAGAG-3’, qSPHK2-F: 5’-TGACCAATAGAAGCAACCGG-3’; qHDAC1-F: 5’-GGATACGGAGATCCCTAATG-3’, qHDAC1-R: 5’-CGTGTTCTGGTTAGTCATATTG-3’; qTET3-F: 5’-TGCGATTGCGTCGAACA-3’, qTET3-R: 5’-TGCGGATCACCCACTTTG-3’. qTNF-α-F: 5’-GCCTCTTCTCCTTCCTGATCGT-3’, qTNF-α-R: 5’- TGAGGGTTTGCTACAACATGGG-3’ [67]; qIL6-F: 5’- AGTGAGGAACAAGCCAGAGC-3’, qIL6-R: 5’- GTCAGGGGTGGTTATTGCAT-3’ [68].

### Luciferase report assay

100 ng pGL3-IFNβ-promoter, pGL3-NF-κB-promoter or the indicated plasmids were co-transfected with 20 ng pRL-TK vector into cells in 24 well plates using LipoD293 [69]. After 24 h, the samples were obtained according to the manufacturer’s instructions (Beyotime, Shanghai, China), and Firefly and Renilla luciferase activities were detected using the Dual Luciferase Reporter Assay kit (Beyotime) according to the indicated protocol.

### Immunofluorescence assay

Cells in confocal dishes were washed with ice-cold PBS, and then fixed with 4% paraformaldehyde for 20 min at room temperature. The cells were then wash with ice-cold PBS for three times (5min/time) again and permeabilized with 1×PBS-T containing 0.2% TritonX-100 for 10 min. Subsequently, cells were blocked with 1×PBS-B containing 0.4% BSA for 30 min at 37°C after washed with PBS. Cells were incubated with the indicated primary antibodies (anti-SPHK2 (R, 17096-1-AP), anti-IAV NP (M, ab128193)) (1:100) overnight at 4°C, followed by Alexa Flour 555, Alexa Flour 647 conjugated secondary antibodies (1:1000) for 1 h. And then DAPI was used for nuclear staining (5 min), 50% glycerin was used for sealing. Images were obtained using a Leica SP8 TCP confocal microscope. Quantitative analyses of IF images were obtained using a 63×objective with LAS X software from three representative images.

### ChIP assay

A549 cells in 6cm plates were fixed with 1% formaldehyde and quenched with 1.25 M glycine. Subsequently, the cells were washed, collected and lysed in 0.5% SDS lysis buffer at 4°C for 30 min. After the cell lysis, the supernatants were obtained and disrupted by Ultrasonic Processor (Scientz). The supernatants were then collected, diluted and incubated with protein A/G magnetic beads (Millipore) at 4°C for 1 h. The indicated antibodies were added for overnight incubation at 4°C. Then protein A/G magnetic beads were added for another two hours, followed by washing (low salt wash buffer, high salt wash buffer, LiCl wash buffer and TE buffer), eluting (Elution buffer: 10% SDS, 1 M NaHCO_3_), and reverse crosslinking processes (incubated with 5 M NaCl at 65°C overnight). The DNA was finally purified and quantified by qRT-PCR. The relative enrichments of promoters are normalized to IgG (-) group, and 2ˆ^-ΔΔCt^ method was used in the calculation. The primers of ChIP-qPCR are as follows: ChIP-qPCR-IFN-β-F: 5’- TTCCTTTGCTTTCTCCCAAGTC-3’, ChIP-qPCR-IFN-β-R: 5’- CAGAGGAATTTCCCACTTTCACTT-3’ [69]; ChIP-qPCR-TGF-β1-F: 5’-CCTGCCGACCCAGCC-3’, ChIP-qPCR-TGF-β1-R: 5’- CTCGCTGTCTGGCTGCT-3’ [36]; ChIP-qPCR-IL6-F: 5’- TCTGCAAGATGCCACAAGGT-3’, ChIP-qPCR-IL6-R: 5’- TGAAGCCCACTTGGTTCAGG-3’ [70].

### Plaque assay

MDCK cells were seeded in 6-well plates to be just 80–90% confluent the following day. A 10-fold serial dilution of samples from virus infection assay were prepared in infection media. Cells were washed, inoculated with 800 μL diluted samples and incubated at 37°C for 1 h. Cells were then washed and overlaid with 2 mL/well of media (2×F12-DMEM (Gibco) containing 2% Agar (Oxoid), 0.3% BSA, 0.1% NaHCO_3_ (Sigma-Aldrich) and 1 μg/mL TPCK-treated trypsin). After a 48–72 h incubation, the cells were fixed with 4% paraformaldehyde for 1 h. The agar layer was then gently removed, and stained with 0.1% crystal violet solution (Sigma-Aldrich). Plaques were subsequently counted and viral titers were expressed as PFU/mL.

## Statistics

All data are shown as mean values and standard deviations of biological replicates (the details are given in the table and figure legends). Statistical significance of the differences between groups was estimated by using two-tailed Student’s t-test, two-tailed Student’s t-test with Welch’s correction, one-way ANOVA or a two-way ANOVA with GraphPad Prism 6. P values was depicted as follows: ns, no significance; *, P<0.05; **, P<0.01; ***, P<0.001; ****, P<0.0001.

## Supporting information

S1 Fig The translocation of SPHK2 in A549 cells after IAV, ZIKV or EV71 infection. A549 cells were uninfected or infected with influenza A/WSN/33 (H1N1) virus (WSN), Zika virus (ZIKV) or Enterovirus 71 (EV71) at an MOI of 0.5 for 24 h. (**A**) Cells were fixed and stained using DAPI for nuclei (Blue), as well as anti-viral protein (IAV NP, ZIKV NS1 or EV71 VP1) antibodies (Green) and anti-SPHK2 antibodies (Red). The cells were visualized by confocal laser scanning microscopy. (**B**) The protein levels of SPHK2 in uninfected, ZIKV or EV71 infected A549 cells were detected by Western blotting, β-actin and Lamin B were used as loading controls for cytoplasmic and nuclear proteins, respectively. Results are representatives of three independent experiments.
(TIF)

S2 Fig The growth rate of SPHK2 overexpressed or knock out A549 cells. (**A**) A549 cells were transfected with pCMV-Myc or Myc-SPHK2, cells with mock transfection were as CTRL, and cell proliferation was assessed by trypan blue exclusion analyses. (**B**) The mRNA and protein levels of SPHK2 in knockout A549 cells were detected separately by qPCR and Western blotting. (**C**) The cell proliferation of A549 cells and the SPHK2 knock out A549 cells (sgSPHK2-1) were monitored by trypan blue exclusion analyses. Results are representatives of three independent experiments.
(TIF)

S3 Fig The growth rate and SPHK2 activity in A549 cells transfected with Myc-SPHK2 (WT) or Myc-SPHK2 mutant (G212E). (**A**) A549 cells were transfected with Myc-SPHK2 (WT) or Myc-SPHK2 mutant (G212E), cells with mock transfection were as CTRL, and cell proliferation was assessed by trypan blue exclusion analyses. (**B**) SPHK2 activity in SPHK2-WT or SPHK2-G212E overexpressed A549 cell normalized to the CTRL is displayed, meanwhile, Myc-tagged SPHK2-WT, SPHK2-G212E protein and endogenous SPHK2 protein levels in SPHK2-WT or SPHK2-G212E overexpressed A549 cell lysates were detected by Western blotting, and β-Actin was as an internal control. Data are means ± SD of three independent experiments. ***, P<0.001.
(TIF)

S4 Fig The mRNA levels of TNF-α and IL6 in SPHK2 overexpressed or knock out A549 cells after IAV infection. (**A**) A549 cells were transfected with pCMV-Myc (-) or SPHK2-expressing plasmids, and 24 h after transfection, cell were infected with WSN virus at an MOI of 0.5. The mRNA levels of TNF-α and IL6 were analyzed by qPCR at 24 hpi, which was normalized to GAPDH. (**B**) A549 cells (WT) or SPHK2 knockout A549 cells (KO) were infected with WSN virus at an MOI of 0.5 for 24 h. The mRNA levels of TNF-α and IL6 were analyzed by qPCR at 24 hpi, which was normalized to GAPDH. Data are means ± SD of three independent experiments. ns, not significant.
(TIF)

S5 Fig SPHK2 activity in shCTRL or shHDAC1 A549 cells infected with IAV and IFN-β promoter activity in Myc-SPHK2 transfected shCTRL or shHDAC1 A549 cells with IAV infection. (**A**) SPHK2 activity in IAV infected and Myc-SPHK2 transfected shCTRL A549 cell lysates or shHDAC1 A549 cell lysates normalized to the CTRL is displayed. (**B**) shCTRL or shHDAC1 A549 cells were transfected with pCMV-Myc, Myc-SPHK2, and then infected with WSN virus at an MOI of 0.5. IFN-β activity was measured by using luciferase reporter assays. Data are means ± SD of three independent experiments. ***, P<0.001; ****, P<0.0001; ns, not significant.
(TIF)

S6 Fig The colocalization analysis of SPHK2 with overexpressed HDAC1 or TET3 after IAV infection. (**A**) A549 cells were transfected with Myc-HDAC1 or Flag-TET3, after 24 h transfection, cells were infected with influenza A/WSN/33 (H1N1) virus (WSN) at an MOI of 0.5 for 24 h. Cells were fixed and stained using DAPI for nuclei (Blue), as well as FITC-anti-influenza A virus NP antibodies (Green), anti-Myc or anti-Flag antibodies (Yellow) and anti-SPHK2 antibodies (Red). The cells were visualized by confocal laser scanning microscopy. (B) shHDAC1 A549 cells were mock-infected or infected with WSN at an MOI of 0.5 for 24 h. Cells were fixed and stained using DAPI for nuclei (Blue), as well as FITC-anti-influenza A virus NP antibodies (Green) and anti-SPHK2 antibodies (Red). The cells were visualized by confocal laser scanning microscopy. Results are representatives of three independent experiments.
(TIF)

S7 Fig The interaction of SPHK2 (FL), SPHK2 mutant (G212E) or its truncated fragments with endogenous HDAC1, TET3 and IFN-β promoter. (**A**) A549 cells transfected with pCMV-Myc, Myc-SPHK2-FL and SPHK2 truncated fragments (Myc-SPHK2-F1, Myc-SPHK2-F2, and Myc-SPHK2-F3) were immunoprecipitated using anti-Myc antibody, then SPHK2-FL, SPHK2-F1, Myc-SPHK2-F2 and SPHK2-F3 proteins in immunoprecipitated complex were analyzed by immunoblotting using Myc-antibody or SPHK2 antibody. (**B**) A549 cells transfected with pCMV-Myc, Myc-SPHK2-FL, Myc-G212E or SPHK2 truncated fragments (Myc-SPHK2-F1, Myc-SPHK2-F2, and Myc-SPHK2-F3) were immunoprecipitated using anti-Myc antibody, and then Myc-tagged SPHK2, HDAC1, and TET3 proteins were analyzed by immunoblotting. (**C**) A549 cells were transfected with pCMV-Myc, Myc-SPHK2-FL, Myc-G212E or SPHK2 truncated fragments, and IFN-β promoter enrichment for Myc-SPHK2-FL, Myc-G212E or SPHK2 truncated fragments was detected by ChIP-qPCR. Data are means ± SD of three independent experiments. *, P<0.05; **, P<0.01.
(TIF)

S8 Fig The TGF-β1 and IL6 promoter enrichment in SPHK2 overexpressed or IAV infected A549 cells. (**A**) A549 cells were transfected with pCMV-Myc, Myc-SPHK2, 24 h post transfection, TGF-β1 and IL6 promoter enrichment for Myc-SPHK2 was detected by ChIP-qPCR, TGF-β1 and IL6 promoter enrichment for TET3 was as a positive control. (**B**) A549 cells were infected with IAV WSN at MOI of 0.5, after 24 h infection, TGF-β1 and IL6 promoter enrichment for SPHK2 was detected by ChIP-qPCR. Data are means ± SD of three independent experiments. ns, not significant.
(TIF)

S9 Fig The interaction of SPHKs with TET3 in A549 cells co-transfected with Flag-tagged TET3, Myc-SPHK1 or Myc-SPHK2. A549 cells co-transfected with Flag-TET3, Myc-SPHK1 or Myc-SPHK2 were immunoprecipitated using anti-Myc antibody, then Myc-tagged SPHK1, SPHK2 and Flag-tagged TET3 proteins were analyzed by immunoblotting. (**B**) HEK293 cells treated with SPHK1 inhibitor SK1-I were transfected with pCMV-Myc, Myc-SPHK2 and then infected with WSN virus at an MOI of 0.5. IFN-β activity was measured by using luciferase reporter assays. Data are means ± SD of three independent experiments. ***, P<0.001.
(TIF)
