# Supplementary figures and images for: Enzymatic independent role of sphingosine kinase 2 in regulating the expression of type I interferon during influenza A virus infection
Source: PLoS Pathog. 2022 Sep 7;18(9):e1010794. doi: 10.1371/journal.ppat.1010794 (PMC9451060; doi:10.1371/journal.ppat.1010794)

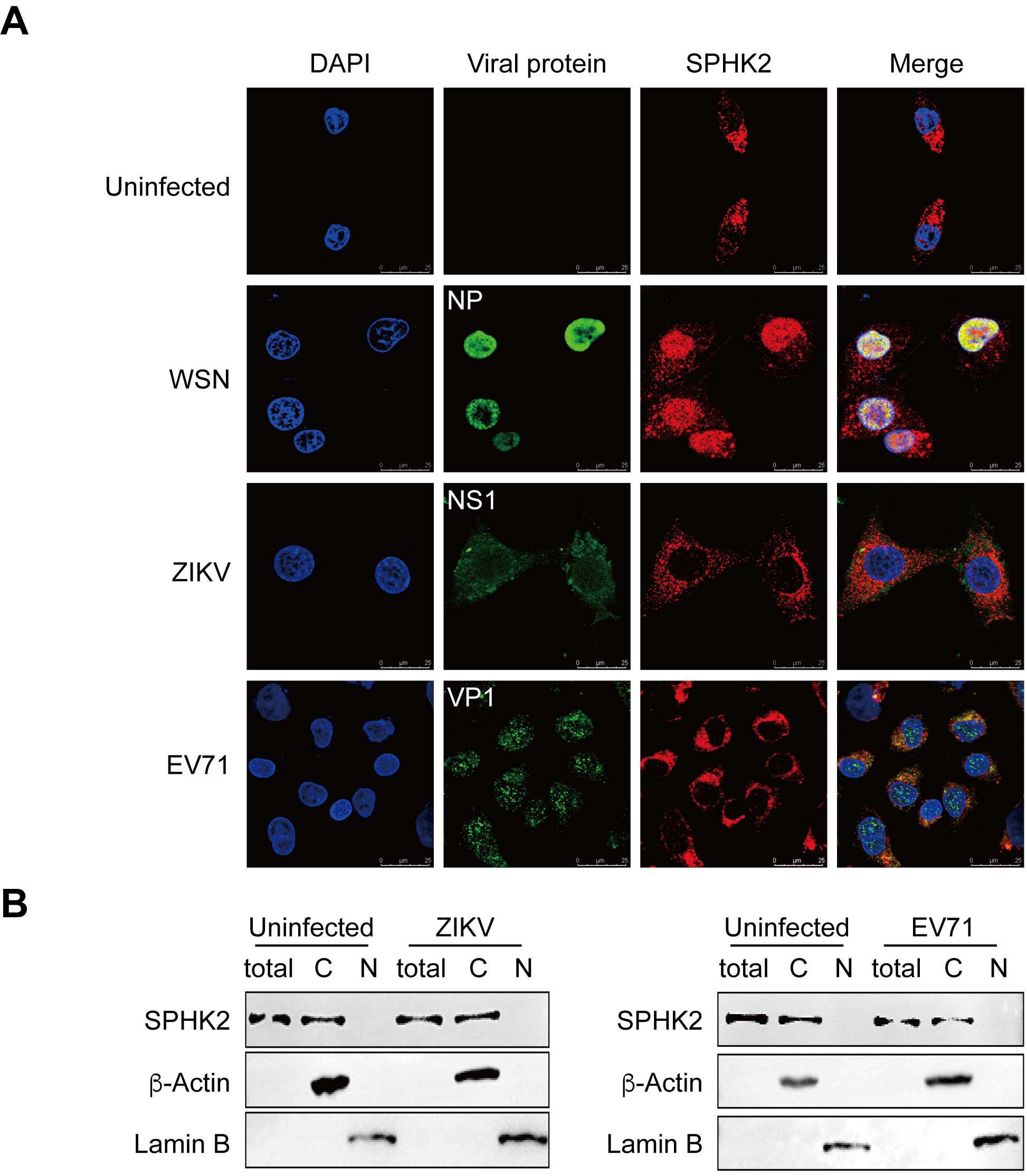

Supplement: S1 Fig — A549 cells were uninfected or infected with influenza A/WSN/33 (H1N1) virus (WSN), Zika virus (ZIKV) or Enterovirus 71 (EV71) at an MOI of 0.5 for 24 h. (A) Cells were fixed and stained using DAPI for nuclei (Blue), as well as anti-viral protein (IAV NP, ZIKV NS1 or EV71 VP1) antibodies (Green) and anti-SPHK2 antibodies (Red). The cells were visualized by confocal laser scanning microscopy. (B) The protein levels of SPHK2 in uninfected, ZIKV or EV71 infected A549 cells were detected by Western blotting, β-actin and Lamin B were used as loading controls for cytoplasmic and nuclear proteins, respectively. Results are representatives of three independent experiments. (TIF) [file ppat.1010794.s001.tif]

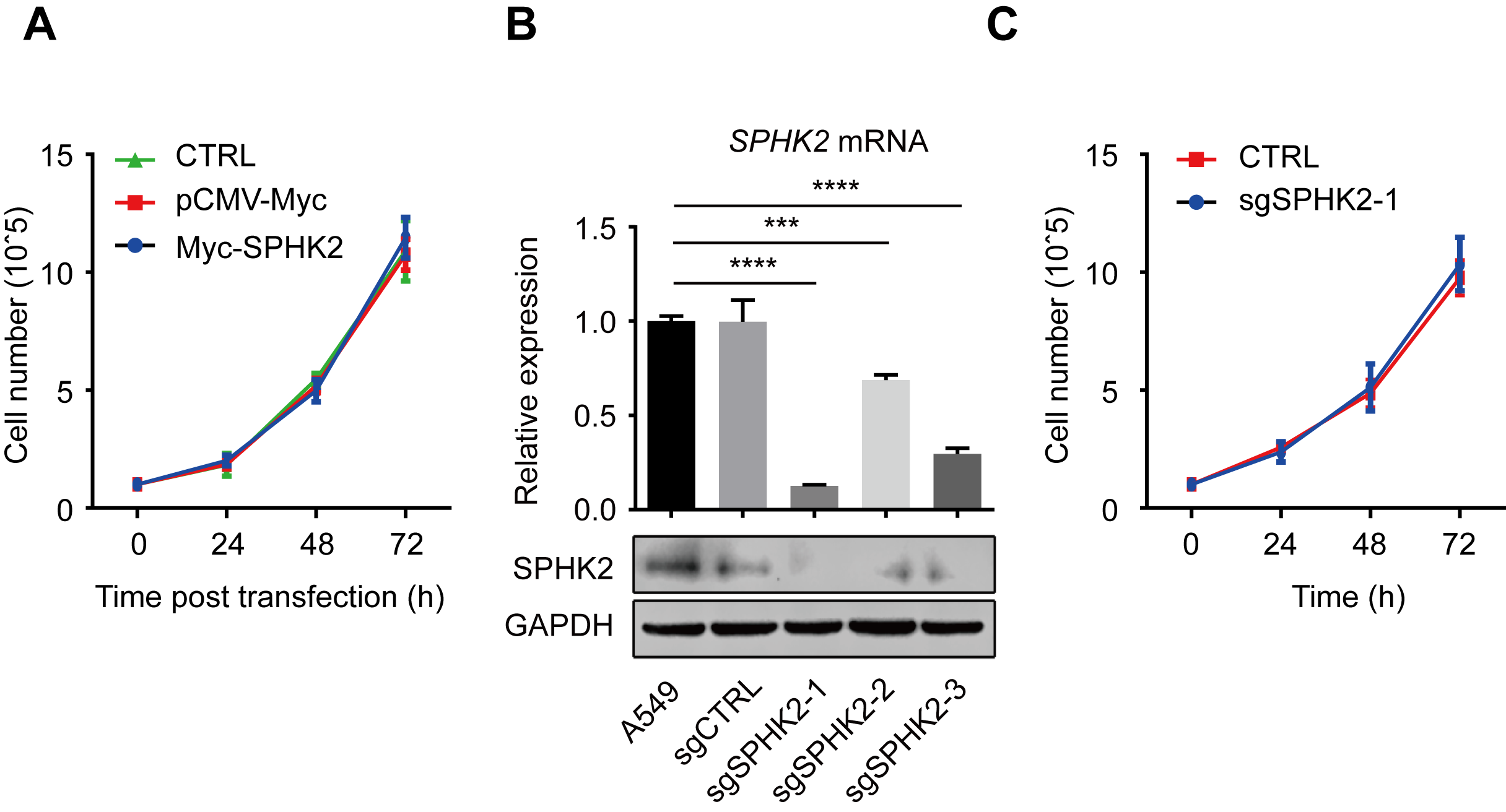

Supplement: S2 Fig — (A) A549 cells were transfected with pCMV-Myc or Myc-SPHK2, cells with mock transfection were as CTRL, and cell proliferation was assessed by trypan blue exclusion analyses. (B) The mRNA and protein levels of SPHK2 in knockout A549 cells were detected separately by qPCR and Western blotting. (C) The cell proliferation of A549 cells and the SPHK2 knock out A549 cells (sgSPHK2-1) were monitored by trypan blue exclusion analyses. Results are representatives of three independent experiments. (TIF) [file ppat.1010794.s002.tif]

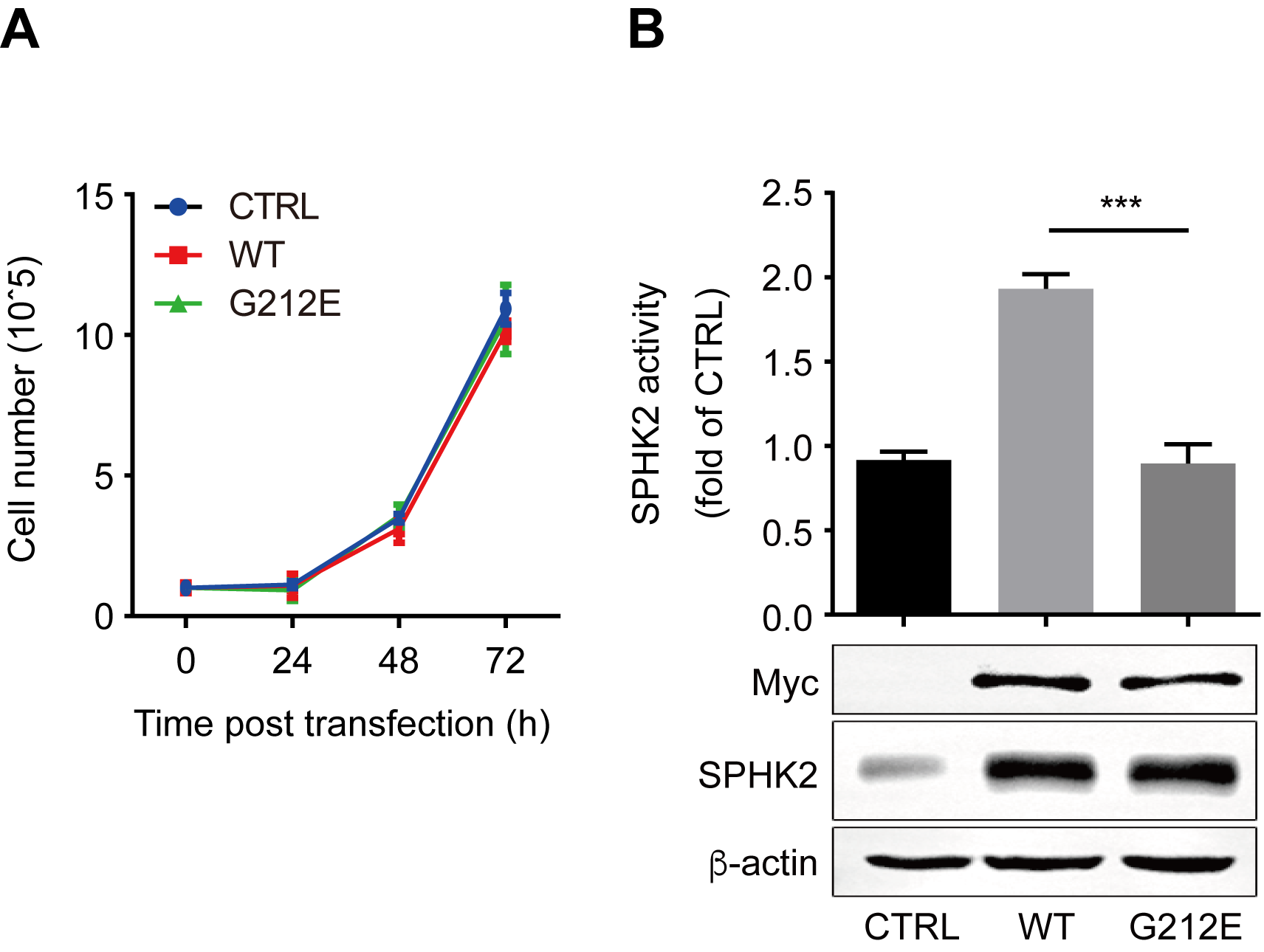

Supplement: S3 Fig — (A) A549 cells were transfected with Myc-SPHK2 (WT) or Myc-SPHK2 mutant (G212E), cells with mock transfection were as CTRL, and cell proliferation was assessed by trypan blue exclusion analyses. (B) SPHK2 activity in SPHK2-WT or SPHK2-G212E overexpressed A549 cell normalized to the CTRL is displayed, meanwhile, Myc-tagged SPHK2-WT, SPHK2-G212E protein and endogenous SPHK2 protein levels in SPHK2-WT or SPHK2-G212E overexpressed A549 cell lysates were detected by Western blotting, and β-Actin was as an internal control. Data are means ± SD of three independent experiments. ***, P<0.001. (TIF) [file ppat.1010794.s003.tif]

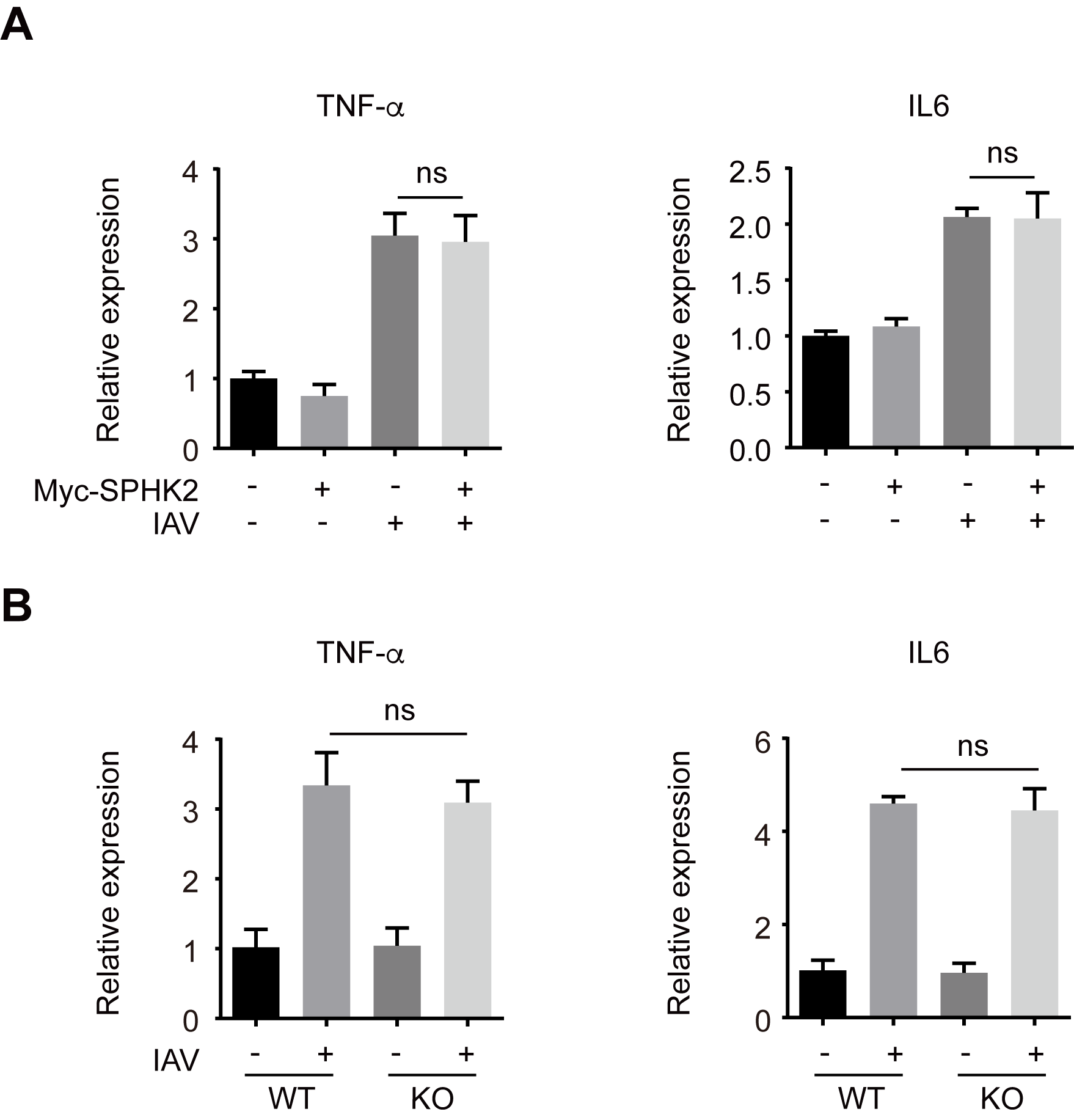

Supplement: S4 Fig — (A) A549 cells were transfected with pCMV-Myc (-) or SPHK2-expressing plasmids, and 24 h after transfection, cell were infected with WSN virus at an MOI of 0.5. The mRNA levels of TNF-α and IL6 were analyzed by qPCR at 24 hpi, which was normalized to GAPDH. (B) A549 cells (WT) or SPHK2 knockout A549 cells (KO) were infected with WSN virus at an MOI of 0.5 for 24 h. The mRNA levels of TNF-α and IL6 were analyzed by qPCR at 24 hpi, which was normalized to GAPDH. Data are means ± SD of three independent experiments. ns, not significant. (TIF) [file ppat.1010794.s004.tif]

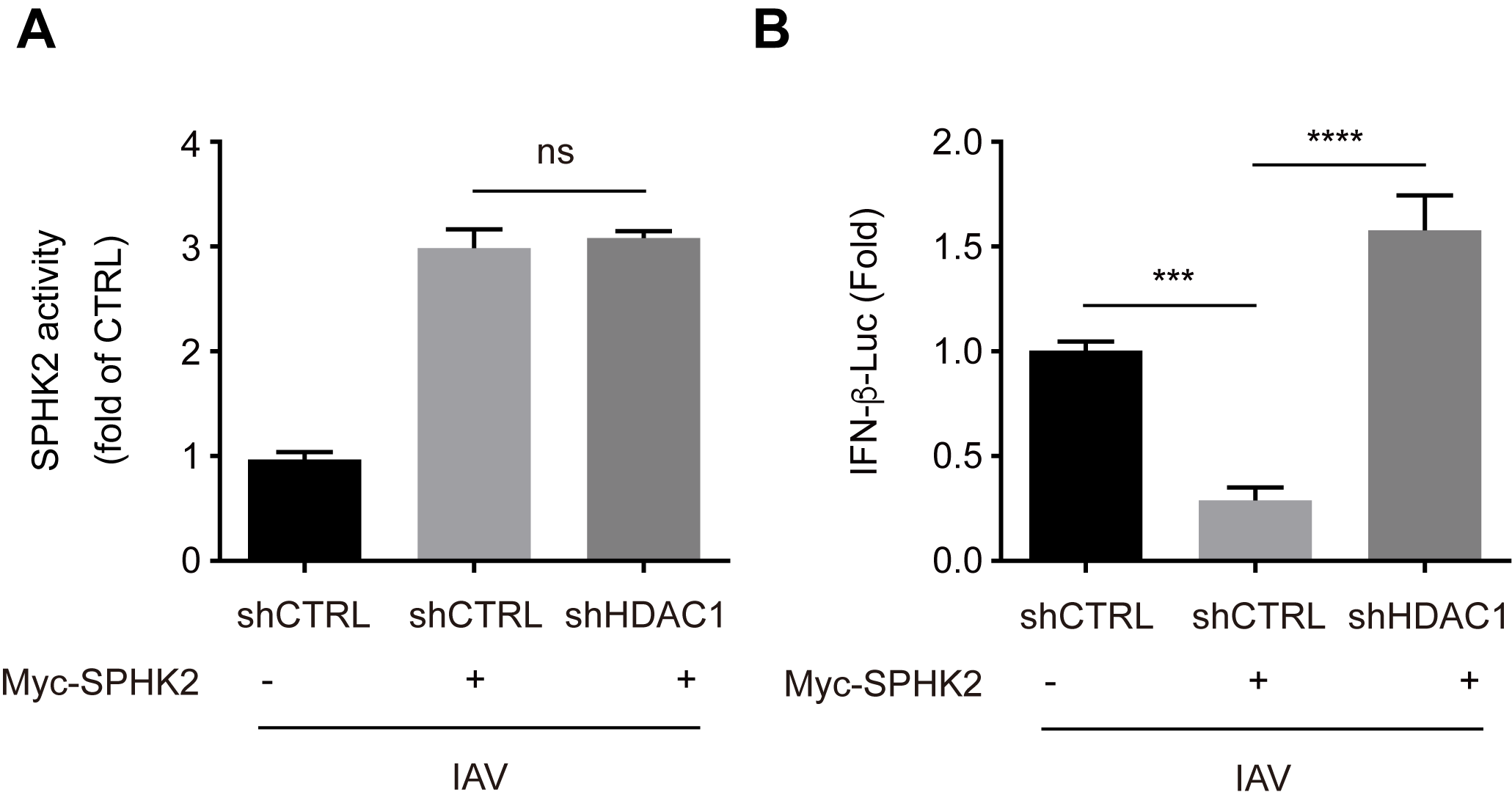

Supplement: S5 Fig — (A) SPHK2 activity in IAV infected and Myc-SPHK2 transfected shCTRL A549 cell lysates or shHDAC1 A549 cell lysates normalized to the CTRL is displayed. (B) shCTRL or shHDAC1 A549 cells were transfected with pCMV-Myc, Myc-SPHK2, and then infected with WSN virus at an MOI of 0.5. IFN-β activity was measured by using luciferase reporter assays. Data are means ± SD of three independent experiments. ***, P<0.001; ****, P<0.0001; ns, not significant. (TIF) [file ppat.1010794.s005.tif]

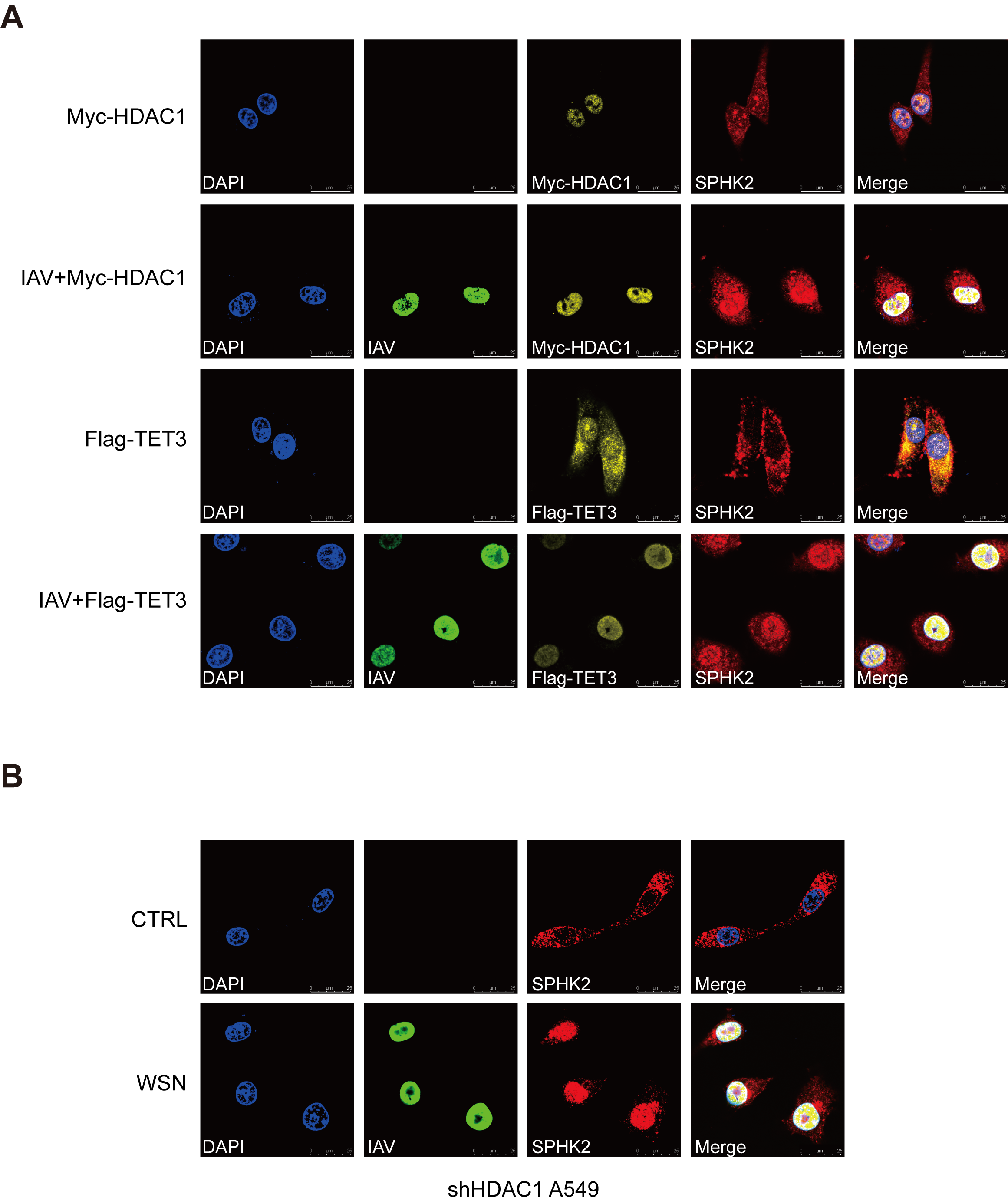

Supplement: S6 Fig — (A) A549 cells were transfected with Myc-HDAC1 or Flag-TET3, after 24 h transfection, cells were infected with influenza A/WSN/33 (H1N1) virus (WSN) at an MOI of 0.5 for 24 h. Cells were fixed and stained using DAPI for nuclei (Blue), as well as FITC-anti-influenza A virus NP antibodies (Green), anti-Myc or anti-Flag antibodies (Yellow) and anti-SPHK2 antibodies (Red). The cells were visualized by confocal laser scanning microscopy. (B) shHDAC1 A549 cells were mock-infected or infected with WSN at an MOI of 0.5 for 24 h. Cells were fixed and stained using DAPI for nuclei (Blue), as well as FITC-anti-influenza A virus NP antibodies (Green) and anti-SPHK2 antibodies (Red). The cells were visualized by confocal laser scanning microscopy. Results are representatives of three independent experiments. (TIF) [file ppat.1010794.s006.tif]

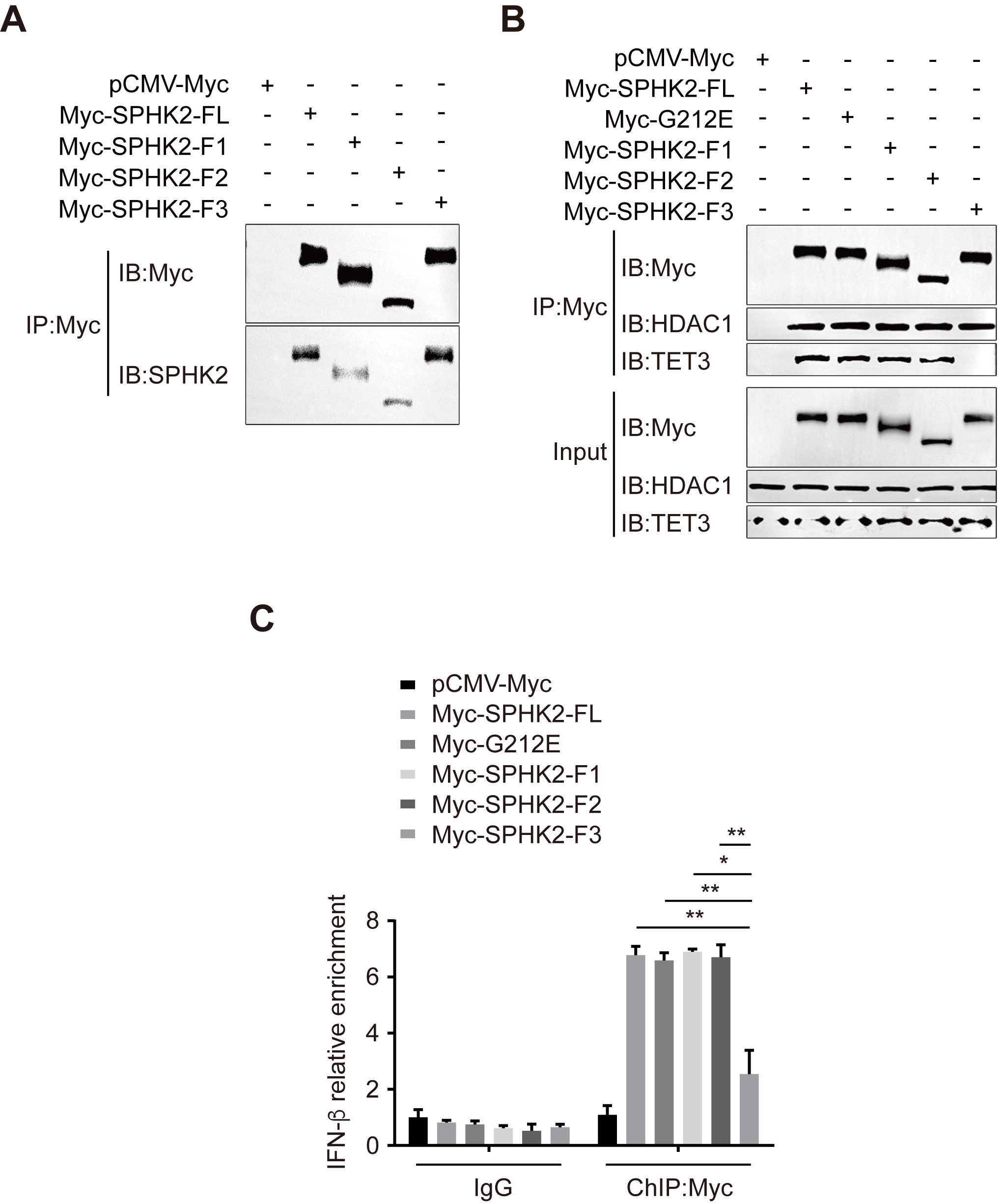

Supplement: S7 Fig — (A) A549 cells transfected with pCMV-Myc, Myc-SPHK2-FL and SPHK2 truncated fragments (Myc-SPHK2-F1, Myc-SPHK2-F2, and Myc-SPHK2-F3) were immunoprecipitated using anti-Myc antibody, then SPHK2-FL, SPHK2-F1, Myc-SPHK2-F2 and SPHK2-F3 proteins in immunoprecipitated complex were analyzed by immunoblotting using Myc-antibody or SPHK2 antibody. (B) A549 cells transfected with pCMV-Myc, Myc-SPHK2-FL, Myc-G212E or SPHK2 truncated fragments (Myc-SPHK2-F1, Myc-SPHK2-F2, and Myc-SPHK2-F3) were immunoprecipitated using anti-Myc antibody, and then Myc-tagged SPHK2, HDAC1, and TET3 proteins were analyzed by immunoblotting. (C) A549 cells were transfected with pCMV-Myc, Myc-SPHK2-FL, Myc-G212E or SPHK2 truncated fragments, and IFN-β promoter enrichment for Myc-SPHK2-FL, Myc-G212E or SPHK2 truncated fragments was detected by ChIP-qPCR. Data are means ± SD of three independent experiments. *, P<0.05; **, P<0.01. (TIF) [file ppat.1010794.s007.tif]

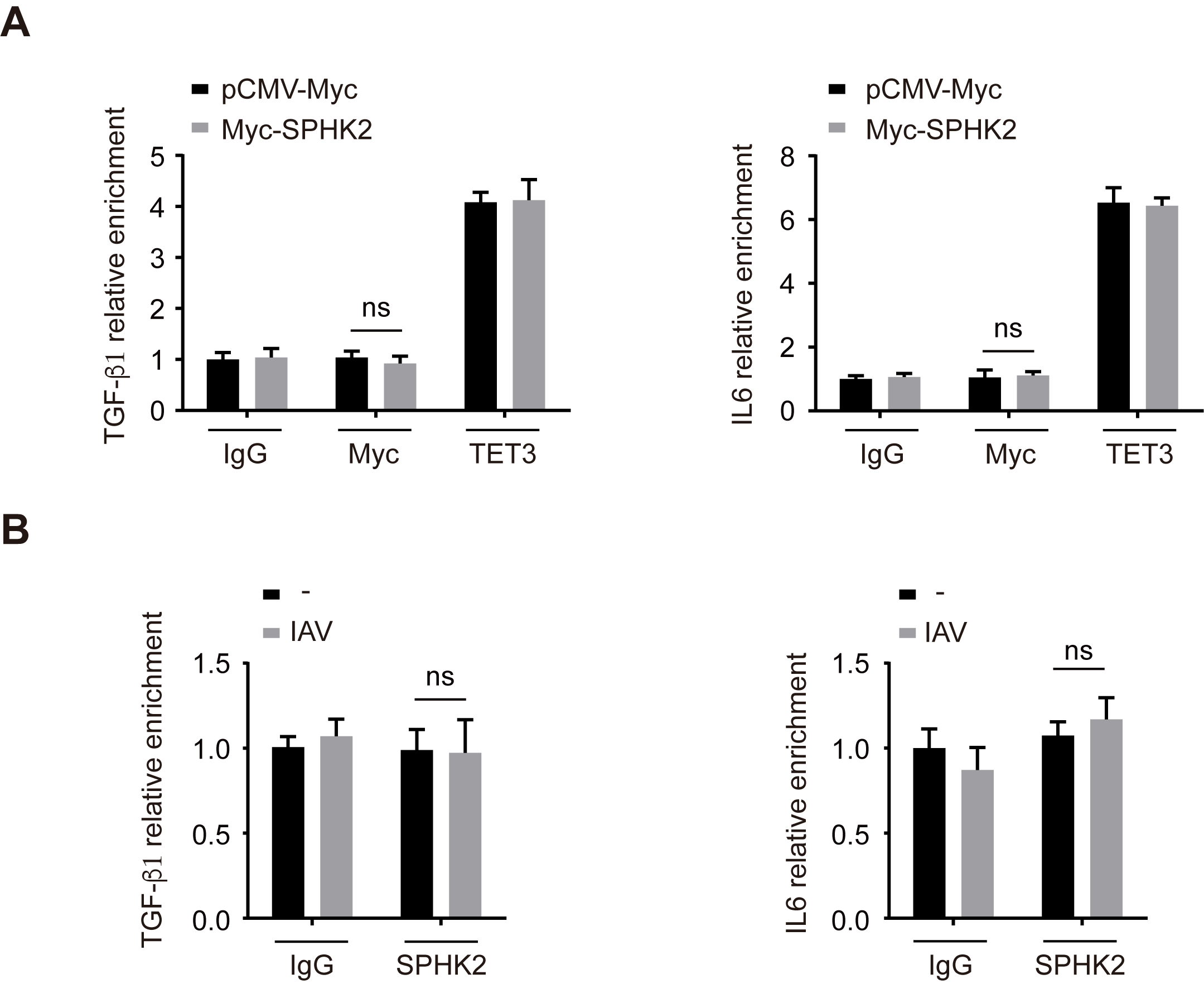

Supplement: S8 Fig — (A) A549 cells were transfected with pCMV-Myc, Myc-SPHK2, 24 h post transfection, TGF-β1 and IL6 promoter enrichment for Myc-SPHK2 was detected by ChIP-qPCR, TGF-β1 and IL6 promoter enrichment for TET3 was as a positive control. (B) A549 cells were infected with IAV WSN at MOI of 0.5, after 24 h infection, TGF-β1 and IL6 promoter enrichment for SPHK2 was detected by ChIP-qPCR. Data are means ± SD of three independent experiments. ns, not significant. (TIF) [file ppat.1010794.s008.tif]

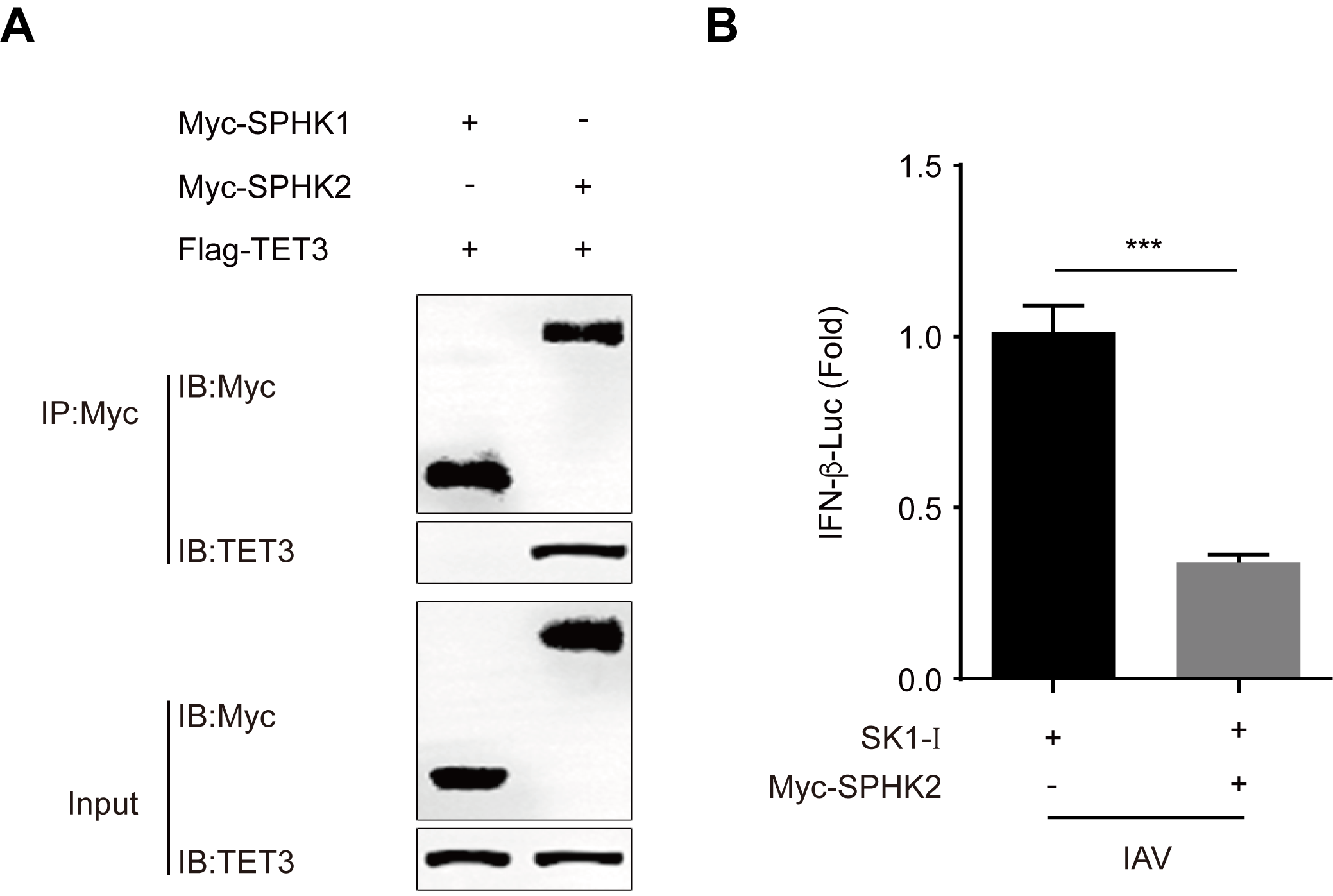

Supplement: S9 Fig — A549 cells co-transfected with Flag-TET3, Myc-SPHK1 or Myc-SPHK2 were immunoprecipitated using anti-Myc antibody, then Myc-tagged SPHK1, SPHK2 and Flag-tagged TET3 proteins were analyzed by immunoblotting. (B) HEK293 cells treated with SPHK1 inhibitor SK1-I were transfected with pCMV-Myc, Myc-SPHK2 and then infected with WSN virus at an MOI of 0.5. IFN-β activity was measured by using luciferase reporter assays. Data are means ± SD of three independent experiments. ***, P<0.001. (TIF) [file ppat.1010794.s009.tif]
